# Methylmalonic Acid, an Aging‐Associated Metabolite, Accelerates Intervertebral Disc Degeneration by Inducing Disc Vascularization via the CCL7/JAK2‐STAT3/VEGF Signaling Axis

**DOI:** 10.1111/acel.70436

**Published:** 2026-03-07

**Authors:** Yuanzhang Jin, Runtian Zhou, Xiaonan Wang, Haifeng Liu, Xiaofeng Zhao, Doudou Jing, Bin Zhao

**Affiliations:** ^1^ Second Hospital of Shanxi Medical University Taiyuan China; ^2^ The Second Affiliated Hospital of Inner Mongolia Medical University Hohhot Inner Mongolia China

**Keywords:** aging, disc vascularization, intervertebral disc degeneration, methylmalonic acid, vascular endothelial growth factors

## Abstract

Intervertebral disc degeneration (IVDD) is an age‐related degenerative spinal disorder, with age as the primary independent risk factor. To investigate the key pathogenic mechanisms of IVDD, we conducted biochemical analyses on IVD specimens from elderly and young groups. In this study, we found that methylmalonic acid (MMA) levels are significantly elevated within the discs of the elderly group, suggesting that MMA may be a critical metabolite involved in aging‐induced IVDD. In in vitro experiments, we observed that MMA treatment of nucleus pulposus cells (NPCs) upregulated the expression of extracellular matrix catabolic markers and downregulated the expression of anabolic markers. Further validation in an in vivo mouse model of needle puncture‐induced IVDD confirmed that MMA accelerates IVDD progression. Mechanistically, we demonstrated that MMA upregulates the expression of C‐C motif chemokine ligand 7 (CCL7) in NPCs. CCL7 acts as a chemoattractant, further enhancing Janus kinase 2/signal transducer and activator of transcription 3 (JAK2/STAT3) signaling transduction, ultimately leading to upregulated vascular endothelial growth factor (VEGF) expression. This promotes abnormal growth of vascular endothelial cells, resulting in disc vascularization. Additional in vivo and in vitro experiments confirmed that disc vascularization is a key progression factor in IVDD. As a rescue strategy, we administered lenvatinib, a VEGF receptor inhibitor, which delayed IVDD progression. Therefore, VEGF and disc vascularization represent a promising therapeutic target for IVDD, offering an innovative approach to addressing IVDD treatment in clinical practice.

## Graphical abstract

Our study reveals that elevated MMA in aged intervertebral discs promotes degeneration by activating the CCL7/JAK2‐STAT3/VEGF signaling axis, which drives pathological vascularization. Therapeutically, the VEGFR inhibitor lenvatinib attenuated this process, identifying disc vascularization as a promising therapeutic target for IVDD.

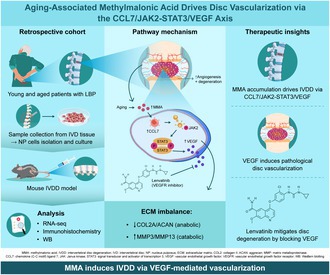

## 1 Introduction

Intervertebral disc (IVD) degeneration (IVDD) is the primary cause of low back pain and restricted lumbar mobility (Oscar et al. 2018; Wen et al. 2023). It is currently the most prevalent spinal degenerative disorder, imposing a substantial economic burden on society (Chen et al. 2024; Xia et al. 2024). A hallmark feature of IVDD is metabolic imbalance, characterized by decreased anabolism and increased catabolism of the extracellular matrix (ECM). This imbalance leads to proteoglycan degradation and a reduction in water content within the IVD (Huang et al. 2023). The development and progression of IVDD result from multifactorial pathological processes, including aging (Wu et al. 2024), excessive mechanical loading (Rannou et al. 2004), obesity (Curic 2020), genetic factors (Feng et al. 2016), and trauma (Bhujel et al. 2022). Aging is the primary independent risk factor for IVDD (Miller et al. 1988). Research has indicated that degenerative changes in human IVDs can begin occurring as early as the second decade of life (Farag et al. 2024). However, the precise pathogenesis underlying aging‐induced IVDD remains incompletely understood (Oscar et al. 2018).

Gomes et al. analyzed serum samples from 30 young and 30 elderly healthy donors, finding that the levels of only three metabolites—phosphoenolpyruvate, quinolinate, and methylmalonic acid (MMA)—were consistently elevated in the serum of elderly donors. MMA is a dicarboxylic acid that is primarily a byproduct of propionate metabolism. Notably, the serum MMA concentration in elderly donors (range: 15–80 μM) was significantly higher than that in young donors (range: 0.1–1.5 μM) (Gomes et al. 2020). Subsequently, Tang et al. (2025) reported consistent findings in a study involving 11,373 participants: individuals with higher serum MMA concentrations tended to be older. Therefore, given its strong correlation with aging, MMA is considered a novel biomarker of aging (Cao et al. 2024). In biological systems, MMA binds to coenzyme A via thioester bonds to form methylmalonyl‐CoA. This compound is then isomerized by methylmalonyl‐CoA mutase (using vitamin B_12_ as a coenzyme) into succinyl‐CoA, thereby entering the tricarboxylic acid (TCA) cycle. MMA accumulation results from increased flux and/or dysregulation of enzymes within this pathway. Currently, the pathological role of MMA has been documented in cardiovascular disease (Wang et al. 2020), kidney disease (Kashtan et al. 1998), and tumor progression (Gomes et al. 2020; Li et al. 2022; Shafer et al. 2025), and may be associated with reactive oxygen species generation and activation of inflammatory pathways. However, a critical gap exists in understanding the role of MMA in IVDD, a disorder similarly driven by degeneration, inflammation, and oxidative stress.

The primary objective of this study was to identify and elucidate the critical metabolites and key molecular regulatory mechanisms underlying the pathogenesis and progression of age‐related IVDD, and ultimately to develop effective strategies for its reversal and treatment.

## 2 Materials and Methods

### 2.1 Patient Data Collection

A retrospective analysis was conducted on patients with low back pain who presented to our institution between January and September 2023. Participants were recruited from the health examination center and the inpatient ward. The following data were collected from each patient: age, height, weight, body mass index, trauma history, smoking history, alcohol consumption history, hypertension, diabetes, osteoporosis, and lumbar spine magnetic resonance imaging (MRI). Three spine surgeons graded the IVDD based on the assessment following Pfirrmann grading system and the modified Pfirrmann grading system (Griffith et al. 2007). Patients were excluded if they had any of the following: concomitant spinal tumors, vertebral deformities or fractures, or a previous history of lumbar spine trauma.

### 2.2 IVD Tissue Collection, Sample Processing, and Disc Cell Culture

Human NP tissue specimens were randomly collected from 30 patients undergoing surgery for lumbar degenerative disc disease (15 males, 15 females; 12 patients aged < 30 years, 18 patients aged > 60 years).

Disc tissue samples from three random young and three random elderly patients were processed for targeted metabolomic analysis. Immediately after retrieval from the operating room, the NP tissue was transferred to a laminar flow hood. The tissue was rinsed with phosphate‐buffered saline (PBS), and excess moisture was carefully blotted using sterile gauze. Samples were temporarily stored at −80°C and batched for subsequent analysis. Targeted metabolomics was performed by Shanghai Bioprofile Technology Co. Ltd. (China).

For primary NP cell isolation, tissue obtained in the operating room was promptly transferred to a laminar flow hood. The tissue was immersed in 75% ethanol for 15 min, rinsed with PBS, and digested with 2 mg/mL type II collagenase overnight. Subsequently, NP cells were extracted and cultured in DMEM/F12 medium containing 15% fetal bovine serum and 1% penicillin–streptomycin and incubated at 37°C in a humidified incubator with 5% CO_2_. NP cells from passages 2–4 were used for in vitro experiments.

### 2.3 Cell Counting Kit‐8 (CCK‐8) Assay

NP cell responses to MMA cytotoxicity and proliferation capacity were quantified via CCK‐8 assay. NP cells were seeded in 96‐well plates at a density of 5 × 10^3^ cells per well. After 24 h, the cells were treated with varying concentrations of MMA (0, 1, 3, 5, 7, 9 mM) for 48 h, with five biological replicates per group. Subsequently, the supernatant was aspirated, and 100 μL of basal medium containing 10% CCK‐8 reagent was added to each well. The plates were then incubated in the dark at 37°C for 2 h. The absorbance of each sample at a wavelength of 450 nm was measured using a microplate reader, and the results were recorded.

### 2.4 Real‐Time Quantitative Polymerase Chain Reaction (RT‐qPCR)

Total RNA was extracted using the TRIzol reagent (Beyotime), and its concentration was measured. A 20 μL reverse transcription system was prepared using 5× Prime Script RT Master Mix (Takara), RNase‐free water (Biosharp), and the extracted mRNA. cDNA was then synthesized via reverse transcription using an Applied Biosystems instrument (Thermo Fisher Scientific). Samples were assessed by RT qPCR on a real time fluorescence quantitative PCR system (QuantStudio 1 Plus). Each sample was run in five technical replicates. GAPDH served as the internal reference control for evaluating the expression of Si CCL7 (Si‐1, Si‐2, Si‐3). Results were analyzed using the 2^−ΔΔCt^ method, which represents the fold change in target gene expression of the experimental group relative to the control group. Table 1 lists the primer sequences.

**TABLE 1:** Sequence of the CCL7 knockout construct.

Si category	Sequence (5′–3′)
CCL7 (h)‐si‐1	GUUGGGAUUAAUACUUCAAtt
	UUGAAGUAUUAAUCCCAACtt
CCL7 (h)‐si‐2	GGGAAGCUGUAAUCUUCAAtt
	UUGAAGAUUACAGCUUCCCtt
CCL7 (h)‐si‐3	CCAAAGCUUUGAACAUUCAtt
	UGAAUGUUCAAAGCUUUGGtt
SiRNA‐NC	UUCUCCGAACGUGUCACGUdTdT
	ACGUGACACGUUCGGAGAAdTdT

### 2.5 Protein Extraction and Western Blotting

NP cells and surgically obtained NP samples were lysed with RIPA buffer. The proteins were separated by sodium dodecyl sulfate polyacrylamide gel electrophoresis and transferred to a membrane, which was blocked using tris‐buffered saline with Tween 20 (TBST) with 5% skim milk for 1 h at room temperature. Then, the membrane was incubated overnight with the primary antibody at 4°C. After three washes with TBST, the membrane was incubated with the secondary antibody at room temperature for 1 h. Protein bands were visualized using a Chem‐iDoc XRS Gel Imaging System (Bio‐Rad Laboratories, Hercules, CA, USA). Glyceraldehyde‐3‐phosphate dehydrogenase (GAPDH) was used as an internal control, and semi‐quantitative analysis was performed using ImageJ software (U.S. National Institute of Health, Bethesda, MD, USA). All antibodies used can be found in Table S1.

### 2.6 Animals and Animal Model Construction

This study was approved by the Ethics Committee of the Second Hospital of Shanxi Medical University. (No. [2025] YX 050). Male C57BL/6J mice aged 8 weeks were obtained from the Animal Center of Shanxi Medical University, housed in an environment maintained at 26°C and 60% humidity with ad libitum access to food and water. First, an MMA injection‐induced accelerated IVDD model was established, according to the intervertebral disc puncture model (F. Li, Shi, et al. 2025). Mice were anesthetized using isoflurane inhalation (300–500 mL/min). The Co5‐6 intervertebral space was identified under X‐ray guidance and marked on the body surface. Following skin disinfection with iodophor, a 26G fine needle was used for puncture to a depth of approximately 1.5 mm. The needle was rotated 180°, held in place for 30 s, and then slowly withdrawn to prevent leakage. Subsequently, the Saline group and the MMA group received an intradiscal injection of 0.2 μL of saline or MMA (5 mM), respectively, via a micro‐syringe (Shanghai Gaoge Industry & Trade, 0.5 mm, 30G) along the original needle track (Han et al. 2025; Zhou et al. 2025). The surgical site was disinfected again post‐operation, and mice were monitored until full recovery from anesthesia. Four weeks post‐surgery, X‐ray and MRI examinations were performed under deep anesthesia, followed by humane euthanasia and collection of coccygeal vertebral specimens. For Stattic intervention, the Sham and MMA groups were treated as described above. The Stattic+MMA group received an injection of 0.2 μL of 5 mM MMA and received a 2‐week course of intraperitoneal injections (IP) of Stattic at a dose of 10 mg/kg, three times a week (P. Li, Liao, et al. 2025; Yokota et al. 2018). To validate the alleviation of IVDD by lenvatinib, mice in the lenvatinib + MMA group received intragastric (IG) of lenvatinib (100 mg/kg/day) starting two weeks prior to modeling (Matsui et al. 2008). The modeling procedure was identical to that for the MMA group. Lenvatinib administration via i.g. was continued for another two weeks post‐modeling. Two weeks later, X‐ray and MRI examinations were conducted under deep anesthesia. Mice were then humanely euthanized. Coccygeal vertebral specimens were collected for further analysis, and tissues from the heart, liver, spleen, lung, and kidney were collected for toxicity analyses (Figure S6B).

### 2.7 Tissue Processing and Staining

Fresh specimens of humans and mouse were fixed using 4% paraformaldehyde for 24 h. The human tissues were directly dehydrated. The mouse disc tissue was decalcified with decalcification solution for 14 days, followed by tissue dehydration, paraffin embedding, and sectioning (5 μm thickness). The slices were stained with hematoxylin and eosin (H&E), Safranin O‐Fast Green (SOFG), and Alcian blue (AB).

### 2.8 Immunohistochemistry (IHC) and Immunofluorescence (IF)

The selected tissue sections were baked at 70°C, followed by deparaffinization. Tissue antigens were retrieved by incubation in a compound digestion solution (Boster, AR022) at 37°C for 45 min; subsequently, endogenous peroxidase activity was blocked by treating the sections with a peroxidase blocking reagent (ZSGB‐BIO, PV‐9000) for 10 min at room temperature. Then, the normal goat serum was used for blocking for 1 h. The slices were incubated overnight at 4°C with the primary antibody, washed in PBS, incubated with the reaction enhancer for 30 min, and then incubated with the secondary antibody at 37°C for 1 h. Immunoreactivity was observed using a DAB chromogenic kit, and the nuclei were stained with hematoxylin after termination.

For IF staining, tissue preparation and pretreatment steps were identical to those used for IHC, with the exception of peroxidase‐based procedures. After washing away the residual primary antibody, the sections were incubated with an HRP‐conjugated secondary antibody for 1 h at room temperature in the dark, followed by incubation with DAPI reagent for 30 min in the dark. Finally, coverslips were sealed using clear nail polish. All antibodies used can be found in Table S1.

### 2.9 X‐Ray and MRI

Four weeks after caudal vertebral needle modeling, anesthetized mice underwent in vivo disc imaging via a small animal in vivo imaging system and an MRI system. Disc Height Index (DHI) and degeneration grade were independently quantified by three blinded diagnostic imaging technicians.

### 2.10 Statistical Analyses

Data analysis and graph generation were performed using GraphPad Prism version 9.5.1 (GraphPad Software, San Diego, CA, USA). All experiments were independently replicated at least three times. Continuous variables are presented as the mean ± standard deviation (mean ± SD), while categorical variables are expressed as frequency (percentage). Following an assessment of variance homogeneity, inter‐group comparisons were performed using a *t*‐test. Comparisons across multiple groups were performed using one‐way ANOVA, with subsequent Tukey's post hoc testing to identify significant differences. Correlation analyses were performed using Pearson's correlation test. A *p*‐value of less than 0.05 was considered statistically significant.

## 3 Results

### 3.1 Aging Is a Key Driver of IVDD, and MMA Is Closely Associated With Aging

**FIGURE 1:**
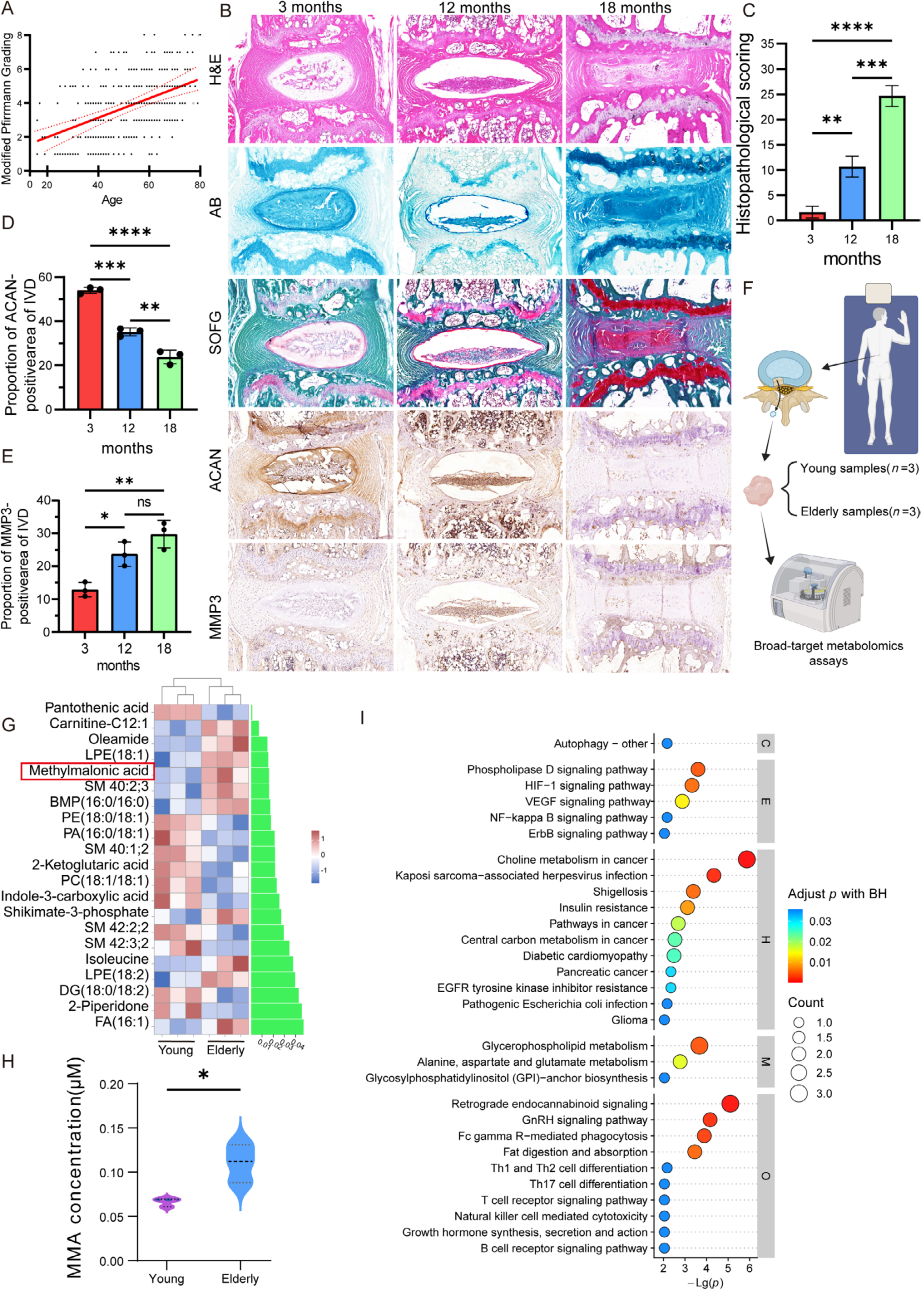
Aging is a key driver of IVDD, and MMA is closely associated with aging. (A) Retrospective regression analysis of age versus modified Pfirrmann grading in patients from Table 1. (B–E) H&E, AB, SOFG staining, and ACAN/MMP3 IHC staining were performed on mice caudal IVD samples at 3, 12, and 18 months. Statistical analysis included histopathological score and immunopositivity rates of ACAN/MMP3 in IVD tissues (*n* = 5). A scale bar of 200 μm (shown in black) applies to all images. (F) Schematic workflow of targeted metabolomics profiling performed on human IVD samples from young and elderly patients (*n* = 3 per group). (G) Heatmap of metabolite abundances. (H) Comparative analysis of MMA levels in IVD tissues between young and elderly patients. (I) KEGG pathway enrichment map. Data are presented as the mean ± SD; *p < 0.05, **p < 0.01, ***p < 0.001, ****p < 0.0001.

A total of 370 eligible individuals were enrolled in this study, including 176 patients in the non‐IVDD group and 194 patients in the IVDD group. The baseline characteristics of the patients are presented in Table 2. The results indicated that age and BMI were significantly associated with IVDD and may serve as risk factors for IVDD, whereas sex, smoking, drinking, hypertension, and diabetes showed no significant correlation with IVDD. To further investigate the relationship between age and IVDD, a univariate logistic regression analysis was performed between age and IVDD grade (Figure 1A). The results demonstrated that increasing age independently correlated with the severity of progressive disc degeneration (*R* ^2^ = 0.2317, *p* < 0.0001), with the IVDD grade escalating with advancing age.

**TABLE 2:** Patient demographics.

	Non‐IVDD (*n* = 176)	IVDD (*n* = 194)	*p*
Female (%)	79 (21.35)	92 (24.86)	0.6765
Male (%)	97 (26.22)	102 (27.57)	
Age (years)	45.93 ± 13.71	59.96 ± 11.71	< 0.0001
BMI (Kg/m^2^)	24.13 ± 3.010	25.33 ± 3.532	0.0005
Smoking (%)	36 (9.73)	41 (11.08)	0.8985
Drinking (%)	32 (8.65)	27 (7.30)	0.3197
Hypertension (%)	29 (7.84)	35 (9.46)	0.7834
Diabetes (%)	24 (6.49)	31 (8.38)	0.5608

To validate these clinical observations, caudal IVD specimens were harvested from C57BL/6 mice aged 3, 12, and 18 months (*n* = 5). Figure 1B presents the H&E, AB, SOFG, and IHC (for COL2A and MMP3) staining results. H&E staining demonstrated progressive age‐dependent degenerative alterations. Compared to discs from young mice (3‐month), discs from aged mice (18‐month) exhibited significant morphological deterioration, including a marked reduction in NP area, transition from elliptical to irregular NP contour, and a loss of demarcation between NP and AF. Histological grading scores increased significantly at 18 months versus 3 months (Figure 1C), confirming a direct correlation between age and degeneration severity in this natural aging model. SOFG and AB staining further revealed an age‐progressive depletion of collagen content and proteoglycan/GAG content within the NP tissue (Figure 1B), indicating accelerated matrix degeneration with advancing age. The IHC analysis demonstrated concomitant age‐dependent molecular shifts, including progressive downregulation of the anabolic ECM marker COL2A and concurrent upregulation of the catabolic enzyme MMP3 (Figure 1B,D,E). Collectively, these histomorphometric and molecular findings corroborate that the severity of IVDD increases intrinsically with biological aging.

Although chronological age is a well‐established driver of natural IVDD, specific age‐associated molecular mediators and pathogenic mechanisms remain incompletely defined. This study aimed to delineate the key biochemical and cellular alterations underlying age‐related disc degeneration and to identify novel therapeutic targets to mitigate disease progression. Therefore, to delineate age‐specific biochemical alterations in the IVD, NP tissues were randomly collected during surgery from three young and three elderly patients; Figure S1A presents their MRI images. An extensive targeted metabolomic analysis was also performed (Figure 1F). A principal component analysis revealed significant metabolic segregation between the young and elderly discs (Figure S1C). Furthermore, pantothenic acid, carnitine‐C12:1, enamide, long‐chain fatty acids (18:1), and MMA were metabolites with large differences between the young and elderly discs based on the metabolic analysis (Figure 1G). Considering that MMA is closely related to age, we measured the absolute content of MMA, finding that it significantly differed between the elderly and young groups; the MMA content in the IVD was more than 1.5 times higher in the elderly group than in the young group (Figure 1H).

MMA and succinate are structural isomers (Figure S1D) but diverge metabolically. Succinate directly enters the TCA cycle, while MMA must bind to coenzyme A to form methylmalonyl‐CoA, which is then isomerized to succinyl‐CoA (vitamin B12 is the coenzyme) by methylmalonyl‐CoA mutase; then, it enters the TCA cycle. This isomerization step is strictly vitamin B_12_ (cobalamin)‐dependent. Consequently, cobalamin deficiency disrupts methylmalonyl‐CoA metabolism, leading to pathological MMA accumulation. Quantification of disc tissue cobalamin levels revealed significantly reduced vitamin B_12_ concentrations in aged discs compared to young controls (Figure S1E). This age‐associated cobalamin depletion likely impairs the conversion of propionate‐derived MMA through the methylmalonyl‐CoA pathway, directly contributing to elevated MMA levels in degenerated discs. A Kyoto Encyclopedia of Genes and Genomes (KEGG) pathway analysis of dysregulated metabolites demonstrated significant enrichment (false discovery rate < 0.05) in the phospholipase D signaling, hypoxia‐inducible factor (HIF)‐1 signaling, vascular endothelial growth factor (VEGF), and inflammatory mediator pathways (Figure 1I). These findings implicate dysregulated HIF‐1α signaling, pathological angiogenesis, and chronic inflammation as key effector mechanisms through which age‐dependent metabolic alterations (particularly MMA accumulation) drive IVDD pathogenesis.

### 3.2 MMA Induces IVDD

**FIGURE 2:**
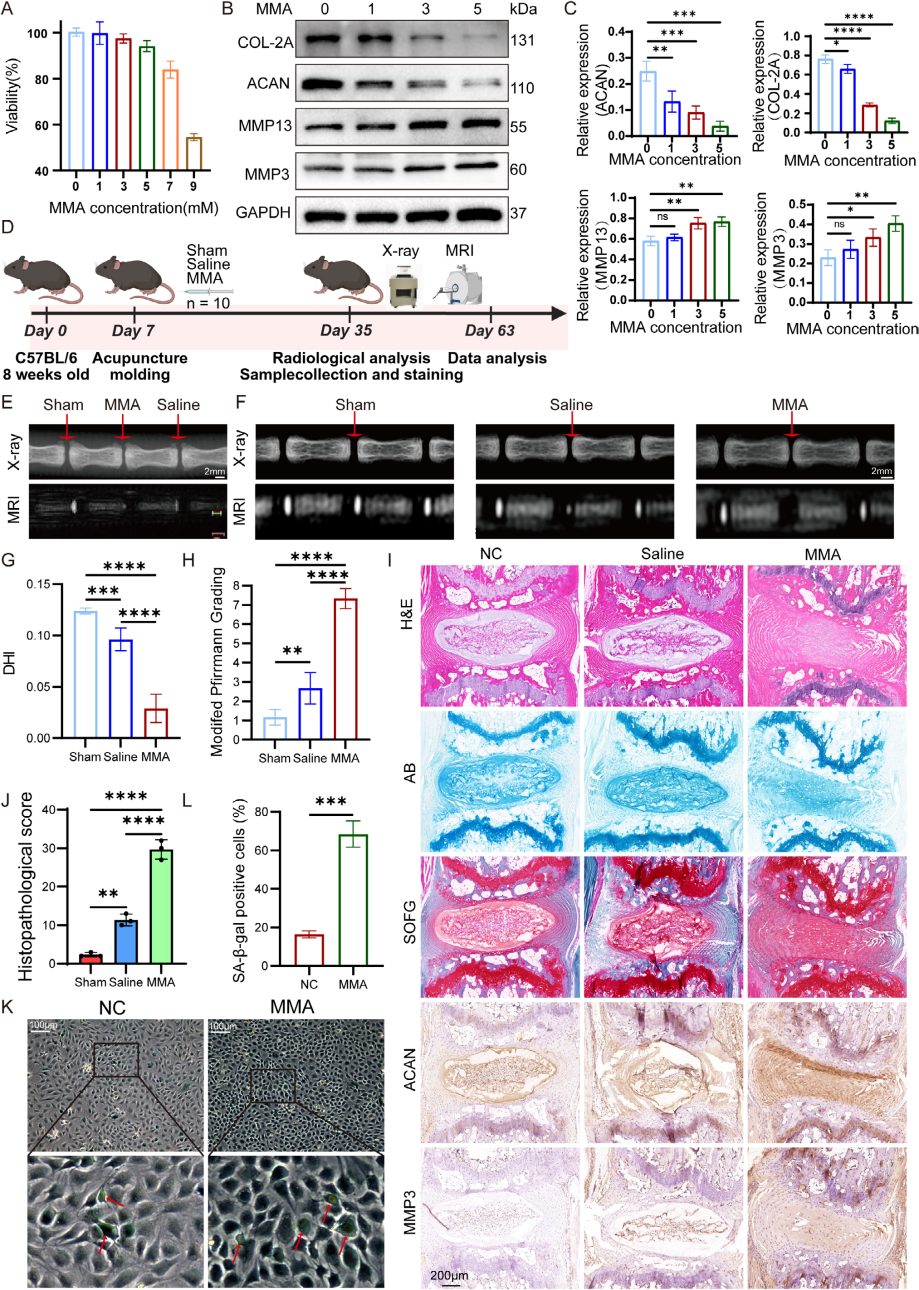
MMA induces IVDD. (A) Initial CCK‐8 assays evaluated the effects of 0–9 mmol/L MMA on NP cell viability over 48 h. (B, C) Western blot analysis and quantification of ACAN, COL2A, MMP3, and MMP13 expression in NP cells treated with 0–5 mmol/L MMA for 48 h, using GAPDH as endogenous control. (D) Schematic diagram of murine caudal vertebral puncture model establishment. (E–H) Representative radiographs and MRI scans of sham, saline, and MMA groups post‐modeling, with quantitative comparisons of DHI and modified Pfirrmann degeneration grades (*n* = 10). The white scale bars in panels E and F indicate a length of 2 mm. (I) Histological staining (H&E, AB, SOFG) and IHC analysis of ACAN/MMP3 in IVD tissues from Sham, Saline, and MMA groups. A scale bar of 200 μm (shown in black) applies to all images. (J) Histopathological score of Sham, Saline, and MMA groups. (K, L) β‐Galactosidase staining of NP cells following negative control or 5 mM MMA intervention, with SA‐β‐gal positive cells quantitative comparisons. The white scale bar represents 100 μm. Data are presented as the mean ± SD; *p < 0.05, **p < 0.01, ***p < 0.001, ****p < 0.0001.

According to Gomes et al. (2020), 5 mM MMA exposure achieves intracellular concentrations at 48 h comparable to those observed following 4‐h treatment with elderly serum. MMA does not easily enter cells on its own. When MMA combines with lipid structures larger than 3 kDa found in serum, its cellular absorption is enhanced. Costa used the MTT test to measure cellular metabolic activity after exposing C6 astrocytoma cells to MMA (0.1–10 mM) for 24 or 48 h. After a 48‐h intervention period, MMA was observed to considerably lower the MTT readings (Costa et al. 2023). Based on the above results, CCK‐8 assays were performed to assess the effects of 0–9 mmol/L of MMA on NP cell viability over 48 h. As a result, 5 mM was selected for subsequent experiments (Figure 2A). This finding aligns with the study by Almeida, C6 astroglial cells were treated with 5 mM MMA (de Souza Almeida et al. 2022), thereby recapitulating physiologically relevant aging conditions. Western blotting of NP cells treated with 0–5 mmol/L of MMA for 48 h revealed dose‐dependent catabolic shifts; the anabolic markers COL2A and aggrecan (ACAN) progressively decreased, while the catabolic enzymes MMP3 and MMP13 significantly increased (*p* < 0.05) (Figure 2B,C). These results confirm MMA‐induced degenerative changes in vitro. β‐Galactosidase staining also showed concentration‐dependent potentiation of cellular senescence (Figure 2K,L).

We used a murine caudal disc (Co3‐Co6) needle‐puncture model to validate these results in vivo using three experimental groups (sham, saline‐injected, and 5 mM MMA‐injected; Figure 2D). Radiographic analyses revealed a significant reduction in the Disc Height Index (DHI) in the MMA group compared to the saline group (*p* < 0.01, Figure 2E,F). An analysis of the DHI and modified Pfirrmann degeneration grades (Figure 2E–H) confirmed the successful induction of IVD degeneration via needle puncture and saline injection. Furthermore, MMA administration significantly exacerbated degenerative progression compared to the saline controls, as evidenced by markedly reduced DHI, substantially elevated Pfirrmann grades approaching maximum severity, and accelerated IVD deterioration. Consistent with the in vitro findings, the histopathological evaluation of the caudal disc specimens (Figure 2I) demonstrated severe degeneration in the MMA group, which was characterized by irregular NP morphology, a pronounced reduction in the NP area, irregular NP morphology, and NP‐AF boundary obscuration. This group also exhibited the highest histological degeneration scores, with statistically significant differences compared to all other controls (Figure 2J), confirming the maximal severity of degeneration. AB and SOFG staining revealed substantial proteoglycan depletion within the NP tissue. Subsequent IHC analyses further validated significant ACAN downregulation and concurrent MMP3 upregulation in MMA‐treated discs (Figure S2B,C), aligning precisely with the in vitro experimental outcomes. Collectively, these results established that MMA potently accelerates IVDD in both in vivo and in vitro models.

### 3.3 MMA Accelerates IVDD via CCL7

**FIGURE 3:**
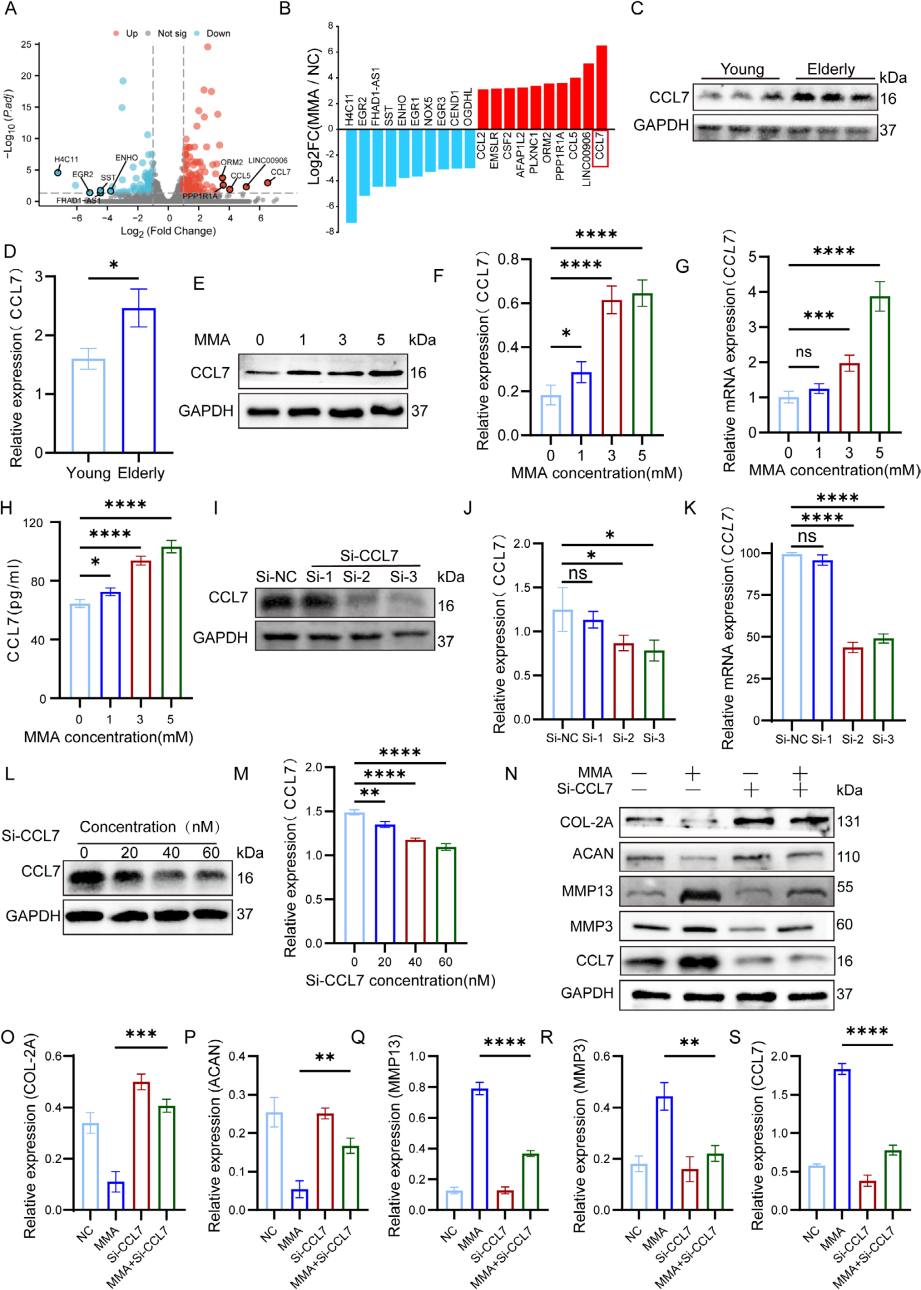
MMA accelerates IVDD via CCL7. (A, B) RNA sequencing results: Volcano plot of differentially expressed genes (DEGs), heatmap of DEGs, and top 10 up‐regulated/down‐regulated genes bar plot. (C, D) Western blot analysis and quantification of CCL7 protein levels in young and aged IVD specimens, with GAPDH as the endogenous control. (E, F) Western blot analysis and quantification of CCL7 protein levels in NPCs treated with 0–5 mM MMA, with GAPDH as the endogenous control. (G, H) mRNA expression levels of CCL7 (measured by RT‐qPCR assay) and concentration levels of CCL7 (measured by ELISA) in groups treated with 0–5 mM MMA intervention on NPCs. (I, J) Western blot analysis and quantification of CCL7 protein levels in NPCs treated with different siRNA knockdown groups, with GAPDH as the endogenous control. (K) mRNA expression levels of *CCL7* in NP cells treated with different siRNA knockdown groups, with GAPDH as the endogenous control. (L, M) Western blot analysis and quantification of CCL7 protein levels in NP cells treated with different concentrations of si‐2, with GAPDH as the endogenous control. (N–S) Western blot analysis and quantification of multiple proteins (ACAN, COL2A, MMP3, MMP13, CCL7) in NP cells treated with NC, MMA, si‐2, or MMA + si‐2, with GAPDH as the endogenous control. Data are presented as the mean ± SD; *p < 0.05, **p < 0.01, ***p < 0.001, ****p < 0.0001.

**FIGURE 4:**
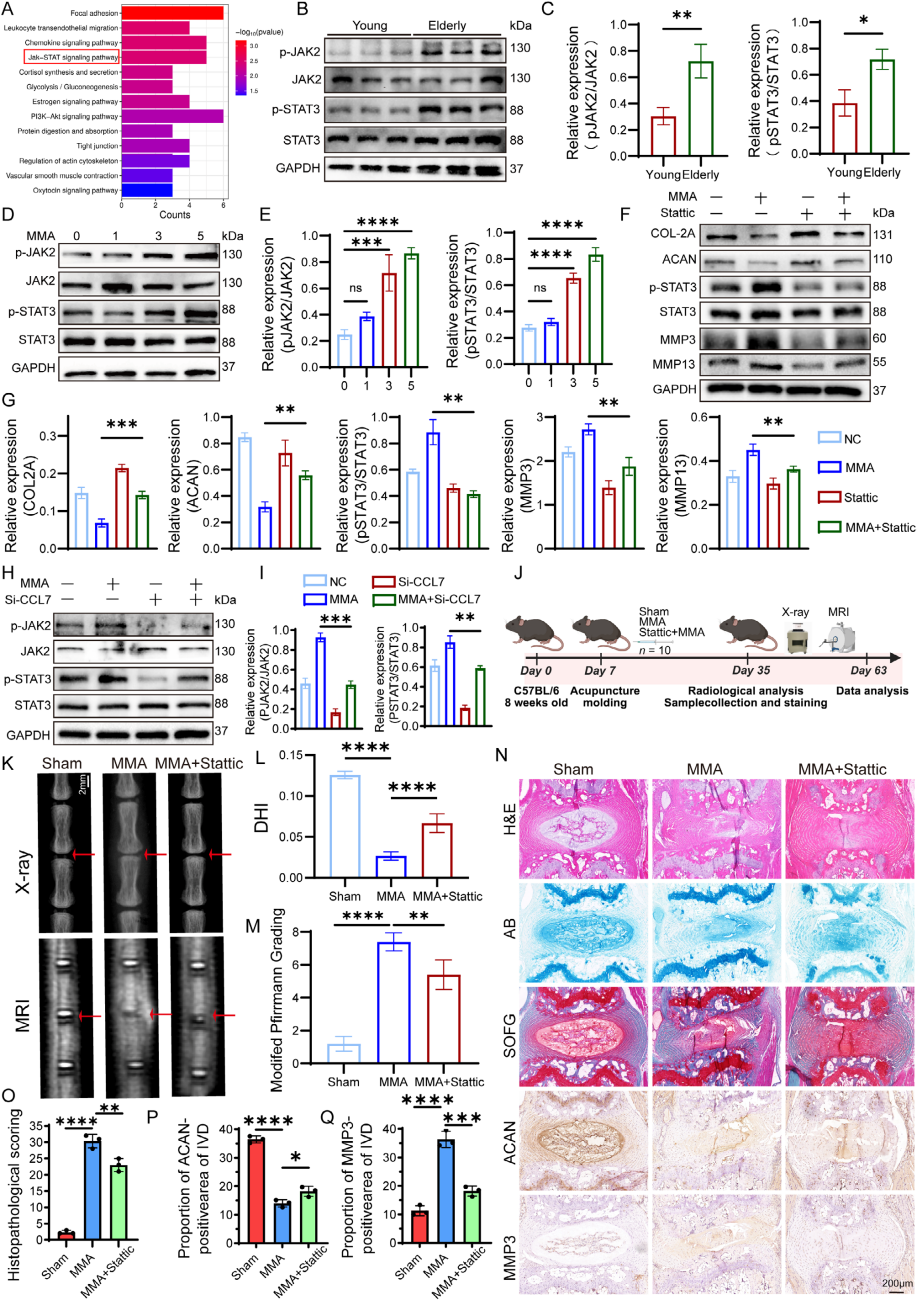
MMA accelerates IVDD through the CCL7‐JAK2/STAT3 signaling pathway. (A) KEGG pathway enrichment analysis of RNA sequencing data. (B, C) Western blot analysis and quantification of pJAK2 and pSTAT3 protein levels in young and elderly IVD tissue specimens, with GAPDH as the endogenous control. (D, E) Western blot analysis and quantification of pJAK2 and pSTAT3 protein levels in NPCs treated with 0–5 mM MMA, with GAPDH as the endogenous control. (F, G) Western blot analysis and quantification of ACAN, COL2A1, MMP3, MMP13, and pSTAT3 protein levels in NPCs treated with NC, MMA, Stattic, or MMA + Stattic, with GAPDH as the endogenous control. (H, I) Western blot analysis and quantification of pJAK2 and pSTAT3 protein levels in NP cells treated with NC, MMA, si‐2, or MMA + si‐2, with GAPDH as the endogenous control. (J) Schematic diagram illustrating the establishment of the needle‐puncture‐induced IVDD model in the mouse caudal spine. Experimental Groups: Sham (control surgery), MMA (local MMA injection), MMA + Stattic (local MMA injection + Stattic inhibitor). (K–M) Representative X‐ray and MRI images of the Sham, MMA, and MMA + Stattic groups post‐modeling. DHI and degeneration grade were quantified for inter‐group comparisons (*n* = 10). A scale bar of 2 mm (shown in white) applies to all images. (N–Q) Histological staining results (H&E, AB, SOFG) and IHC staining for ACAN and MMP3 in IVD tissues from Sham, Saline‐injected control, and MMA groups. Statistical analysis of histological scores and the percentage of ACAN‐positive and MMP3‐positive cells was performed. A scale bar of 200 μm (shown in black) applies to all images. Data are presented as the mean ± SD; *p < 0.05, **p < 0.01, ***p < 0.001, ****p < 0.0001.

RNA sequencing from NPs isolated from the control and 5 mM MMA‐treated groups identified 149 upregulated and 67 downregulated genes (Figure 3A). Figure S3A shows the hierarchical clustering of the differentially expressed genes. An analysis of the top 10 upregulated and downregulated genes (Figure 3B) revealed a significant elevation in the CC chemokine family members: *CCL2*, *CCL5*, and *CCL7*. The KEGG pathway analysis further implicated chemokine signaling (Figure 4A), with *CCL7* exhibiting the most pronounced upregulation, suggesting its critical role in the pathogenesis of IVDD. The known CCL7 receptors (CCR1, CCR2, CCR3, and CCR5) mediate distinct functions; CCR1/CCR3 promote endothelial cell migration, while CCR2/CCR5 drive monocyte mobilization. CCR2 activation specifically triggers G protein‐coupled signaling cascades (phosphoinositide 3‐kinase/Protein kinase B [PI3K/AKT], mitogen‐activated protein kinase [MAPK]/p38, and Janus kinase [JAK]/signal transducer and activator of transcription 3 [STAT3]). Validation studies demonstrated elevated CCL7 protein levels in elderly human discs using western blotting (Figure 3C,D). Western blotting, qRT‐PCR, and enzyme‐linked immunosorbent assays confirmed that dose‐dependent MMA exposure in NPCs upregulated CCL7 (Figure 3E–H). In order to clarify the specific role of *CCL7*, we conducted gene knockout experiments and designed three validated single interfering RNAs (Si‐RNA) (Table 2). Firstly, we verified the knockdown efficiency of the three types of Si‐RNA through WB and PCR. Both Si‐2 and Si‐3 demonstrated significant knockdown effects, with Si‐2 showing a knockdown efficiency of 55%, which was slightly higher than that of Si‐3 (Figure 3I–K). Therefore, Si‐2 was ultimately selected. Subsequently, we determined the usage concentration of Si‐2 to be 40 nM through WB (Figure 3L,M). When applied to MMA‐treated NPCs, it specifically upregulated ACAN/COL2A expression and downregulated MMP3/MMP13 levels (Figure 3N–S), confirming the pivotal role of CCL7 in mediating MMA‐induced disc degeneration.

### 3.4 MMA Accelerates IVDD Through the CCL7‐JAK2/STAT3 Signaling Pathway

The KEGG pathway analysis (Figure 4A) revealed that focal adhesion, leukocyte transendothelial migration, chemokine signaling pathways, and the JAK2‐STAT3 pathway play significant roles in MMA‐induced IVDD. Given that CCL7 triggers various G protein‐mediated intracellular signaling cascades upon CCR2 activation, such as the PI3K/AKT, MAPK, and JAK/STAT3 pathways, we hypothesized that the CCL7‐JAK2/STAT3 signaling pathway critically contributes to MMA‐induced IVDD. Initial analysis of human IVD tissues demonstrated significantly elevated pJAK2 and pSTAT3 expression in the elderly group (Figure 4B,C), indicating the JAK2‐STAT3 pathway plays a key role in aging‐associated IVDD. Subsequent western blot analyses of pJAK2 and pSTAT3 levels in NPCs treated with increasing MMA concentrations (Figure 4D,E) showed a marked concentration‐dependent increase, with significant differences observed at 3 and 5 mM, with a greater effect at 5 mM.

To confirm the functional role of the JAK2‐STAT3 pathway in MMA‐induced IVDD, we employed Stattic, a specific STAT3 phosphorylation inhibitor that suppresses phosphorylation at the Y705 and S727 residues, thereby inactivating STAT3. Based on John's report (McMurray 2006), CCK‐8 assays were used to determine cell viability after 24‐ and 48‐h treatment with various concentrations of Stattic (0, 2.5, 5, 7.5, and 10 μM). As a result, 2.5 μM for 48 h was selected for subsequent assays (Figure S3B). Validation of NPCs treated with 5 mM MMA and Stattic via western blotting confirmed the inhibition of STAT3 phosphorylation. Furthermore, compared to the MMA‐only group, co‐treatment with Stattic significantly ameliorated anabolic markers (COL2 and ACAN), reduced catabolic markers (MMP3 and MMP13), and alleviated disc degeneration (Figure 4F,G), reiterating the critical role of JAK2‐STAT3 in IVDD. To verify the relationship between CCL7 and the JAK2/STAT3 pathway, we knocked down *CCL7* in NPCs. Western blotting results showed that *CCL7* knockdown significantly reduced the basal and MMA‐induced elevations in p‐JAK2/p‐STAT3 expression (Figure 4H,I). Subsequent in vivo experiments involved the administration of MMA and Stattic. As shown in the pattern diagram (Figure 4J), on Day 7, the mice underwent needle puncture under X‐ray guidance and received sham, MMA, or MMA + Stattic treatments. On Day 35, radiography and MRI were performed under anesthesia, followed by the collection of coccygeal specimens for histological staining. X‐ray and MRI results (Figure 4K–M) showed that the MMA + Stattic group maintained a greater DHI on X‐ray and a residual high signal in the NP region on MRI compared to the MMA group, indicating significantly milder disc degeneration. H&E staining (Figure 4N) showed near‐complete loss of structure in the MMA group, with vacuolated cells and indistinct NP‐AF boundaries, whereas the MMA + Stattic group retained irregular NP cells with clearer NP‐AF demarcation; significantly better histopathological score supported this result (Figure 4O). AB and SOFG staining (Figure 4N) demonstrated greater proteoglycan retention within the NP of the MMA + Stattic group than within the MMA group. IHC for ACAN and MMP3 further confirmed higher ACAN and lower MMP3 expression in the MMA + Stattic group (Figure 4P,Q), indicating a reduced degenerative state. Collectively, these results establish the pivotal role of the JAK2‐STAT3 pathway in IVDD and demonstrate that Stattic intervention effectively mitigates the progression of disc degeneration.

### 3.5 MMA Induces IVD Vascularization and Degeneration Through the CCL7‐JAK2/STAT3‐VEGF Axis

**FIGURE 5:**
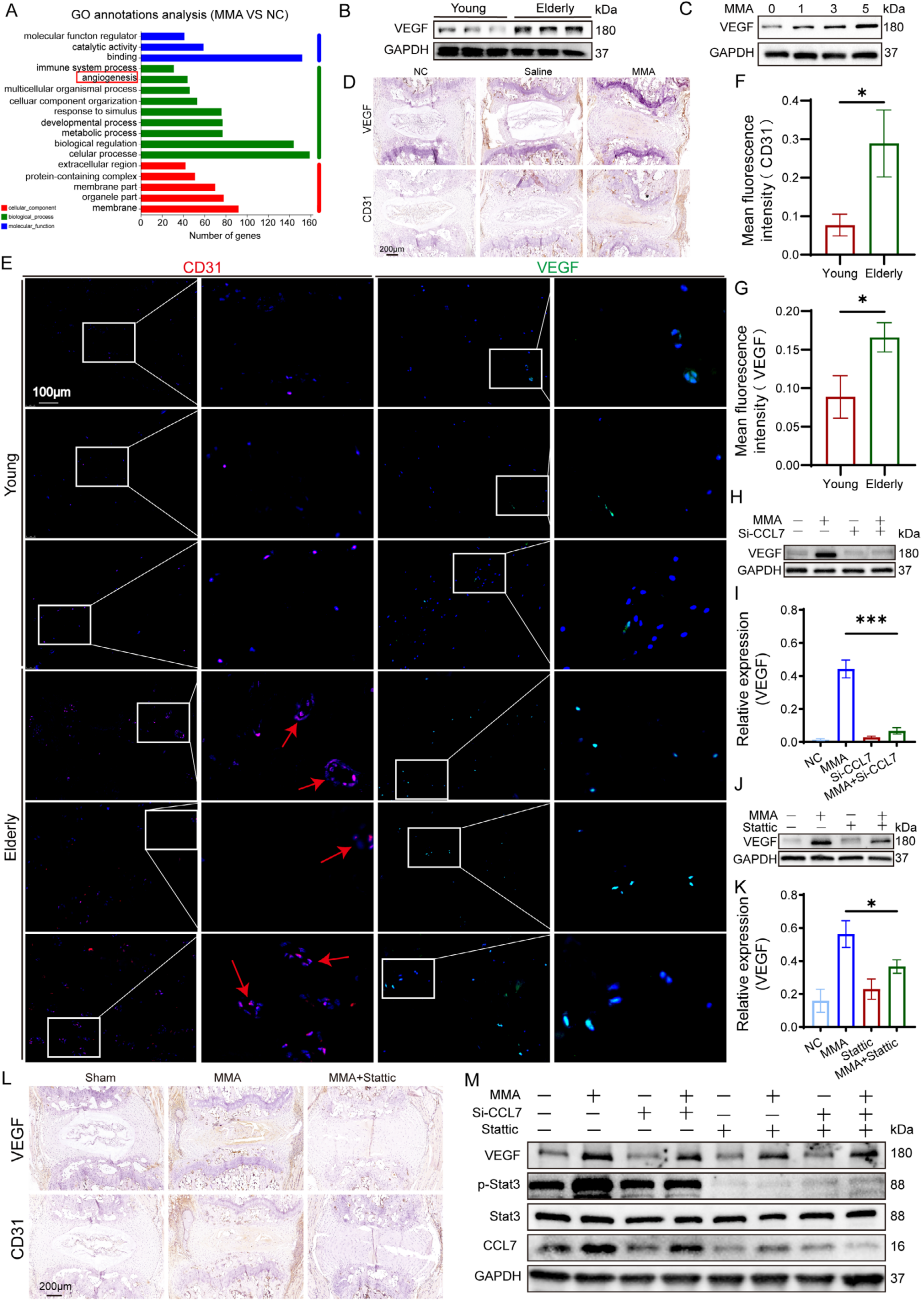
MMA induces IVD vascularization and degeneration through the CCL7‐JAK2/STAT3‐VEGF axis. (A) GO enrichment analysis of RNA sequencing data. (B) Western blot analysis and quantification of VEGF protein levels in young and elderly IVD specimens, with GAPDH as endogenous control. (C) Western blot analysis and quantification of VEGF protein levels in NPCs treated with 0–5 mM MMA, with GAPDH as the endogenous control. (D) Representative IHC staining of VEGF and CD31 in IVD tissues from Sham, Saline, and MMA groups. A scale bar of 200 μm (shown in black) applies to all images. (E–G) IF staining of VEGF and CD31 in young and aged IVD specimens. Statistical analysis of VEGF‐positive cells and CD31‐positive vascular endothelial cells was performed. A scale bar of 100 μm (shown in white) applies to all images. (H, I) Western blot analysis and quantification of VEGF protein levels in NPCs treated with NC, MMA, si‐2, or MMA + si‐2, with GAPDH as endogenous control. (J, K) Western blot analysis and quantification of VEGF protein levels in NP cells treated with NC, MMA, Stattic, or MMA + Stattic, with GAPDH as endogenous control. (L) Representative IHC staining of VEGF and CD31 in IVD tissues from Sham, MMA, and MMA + Stattic groups. A scale bar of 200 μm (shown in black) applies to all images. (M) Western blot analysis and quantification of VEGF, pSTAT3, MMP3, and CCL7 protein levels in NP cells treated with NC, MMA, si‐2, MMA + si‐2, Stattic, MMA + Stattic, si‐2 + Stattic, or MMA + si‐2 + Stattic, with GAPDH as endogenous control. Data are presented as the mean ± SD; *p < 0.05, **p < 0.01, ***p < 0.001, ****p < 0.0001.

A Gene Ontology analysis implicated the immune response, metabolic processes, and angiogenesis in MMA‐induced IVDD (Figure 5A), while prior metabolomic KEGG enrichment (Figure 1J) implicated the HIF‐1 and VEGF pathways, suggesting that VEGF is a pivotal mediator of disc degeneration. Western blotting of human NP tissues demonstrated significantly elevated VEGF protein levels in elderly versus young discs (Figures 5B and S4B). We conducted a Western Blot analysis on NPCs treated with MMA, and found that as the concentration of MMA increased, the expression level of VEGF also rose (Figures 5C and S4C). This finding was recapitulated in vivo using the murine caudal disc puncture model, where IHC of disc specimens (Figures 5D and S4D,E) showed markedly elevated VEGF and CD31 expression in MMA‐treated groups, with substantial VEGF accumulation in the AF and NP vacuolated regions and widespread CD31‐positive staining, particularly near the endplates and outer annular regions. IF examination of VEGF and CD31 in another set of samples (Figure 5E–G) revealed marked expression of VEGF and CD31 in the NP of elderly patients. Moreover, abundant CD31‐stained circular‐shaped blood vessels were prominently observed in the NPCs of older patients, indicating the presence of numerous mature circular vessels with significantly greater vascular density compared to those of younger patients. Murine validation via IHC confirmed age‐dependent upregulation of VEGF/CD31 (Figure S4F–H). Then, the supernatant from MMA‐treated NPSCs was mixed with the culture medium of human umbilical vein endothelial cells (HUVECs) for co‐culture. Scratch wound healing and tube formation assays were performed to evaluate the pro‐angiogenic capacity of the conditioned medium from MMA‐stimulated NPSCs. In the scratch assay, the gap was completely closed in the MMA group within 36 h, demonstrating a significantly enhanced migratory ability of HUVECs cultured with the conditioned medium from the MMA group (Figure S5A,B). In the tube formation assay, a greater number of vascular‐like structures and branch points, and longer capillary length were observed in the MMA group, indicating a markedly superior tube‐forming capability of the HUVECs (Figure S5C–E). Collectively, these findings establish that MMA promotes VEGF‐mediated disc vascularization, leading to degeneration.

To validate the relationship between the CCL7‐JAK2/STAT3 pathway and VEGF in MMA‐induced IVDD, we knocked down *CCL7* using small interfering RNA (si‐CCL7) and inhibited Stattic in NPCs, followed by a western blot analysis (Figure 5H–K). Both interventions significantly attenuated MMA‐induced VEGF elevation. In vivo confirmation via IHC of murine caudal discs revealed that Stattic administration substantially reduced VEGF and CD31 expression (Figures 5L and S5F,G). Finally, combination interventions (e.g., MMA + si‐CCL7 + Stattic) in NPCs followed by western blotting (Figure 5M) conclusively verified the entire CCL7‐JAK2/STAT3‐VEGF signaling axis, ultimately demonstrating its critical role in the pathogenesis of MMA‐driven disc degeneration.

### 3.6 Angiogenesis Inhibitors Attenuate IVDD

**FIGURE 6:**
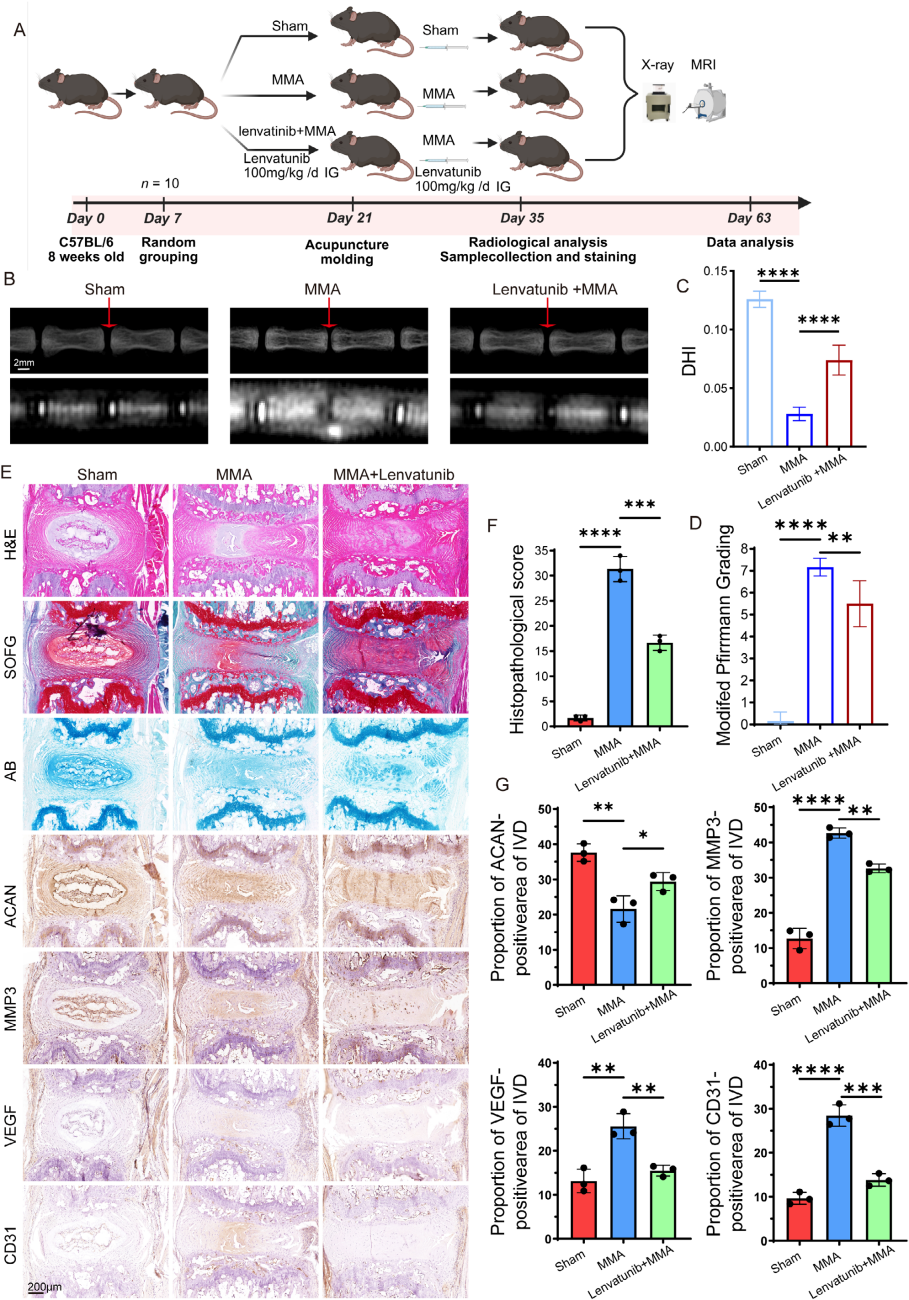
Angiogenesis inhibitors attenuate IVDD. (A) Schematic diagram of the needle‐puncture‐induced IVDD model in the mouse caudal spine. (B–D) Representative X‐ray and MRI images of the Sham, MMA, and MMA + Lenvatinib groups post‐modeling. DHI and degeneration grade were quantified for inter‐group comparisons (*n* = 10). A scale bar of 2 mm (shown in white) applies to all images. (E) Representative staining images: H&E, AB, SOFG and IHC for ACAN, MMP3, VEGF, and CD31 in Sham, MMA, and MMA + Lenvatinib groups. A scale bar of 200 μm (shown in black) applies to all images. (F, G) Statistical analysis of histopathological score and the percentage of ACAN‐positive, MMP3‐positive, VEGF‐positive, and CD31‐positive cells. Data are presented as the mean ± SD; *p < 0.05, **p < 0.01, ***p < 0.001, ****p < 0.0001.

In this study, we established that VEGF‐mediated disc vascularization is critical in IVDD. According to the study (Matsui et al. 2008), lenvatinib treatment more potently inhibits angiogenesis than bevacizumab in the MDA‐MB‐231 model. By comparing the efficacy of lenvatinib, imatinib, and VEGF‐neutralizing antibodies in the H146 xenograft model, Junji Matsui (Matsui et al. 2008) found that at 100 mg/kg, E7080 reduces microvessel density more effectively than anti‐VEGF antibodies and imatinib treatment. Therefore, to further elucidate the functional contribution of vascularization, we validated this mechanism in vivo using lenvatinib, a broad‐spectrum VEGF inhibitor. Figure 6A presents the experimental group schematic. Group 3 received lenvatinib (100 mg/kg/d via intragastric administration) for two weeks prior to uniform puncture modeling, and Groups 2 and 3 received MMA injections. Group 3 continued lenvatinib treatment for an additional 2 weeks. On the 35th day, X‐ray and MRI examinations revealed that the lenvatinib‐treated group (Group 3) had significantly higher DHI and significantly lower modified Pfirrmann grades than the MMA group (Group 2) (Figure 6B–D). Histological staining of the collected specimens (Figure 6E) demonstrated residual NP tissue with partially discernible NP‐AF boundaries in Group 3 versus Group 2, accompanied by lower histopathological score (Figure 6F). AB and SOFG staining confirmed residual proteoglycans in the NP region of mice in Group 3 compared to the severe depletion observed in Group 2 (Figure 6E). Subsequent IHC assays for ACAN, MMP3, VEGF, and CD31 (Figure 6G–J) revealed higher ACAN expression and lower MMP3 levels in the discs from Group 3, indicating a more balanced anabolic/catabolic metabolism and milder degenerative progression, consistent with the imaging findings. The vascularization markers (VEGF and CD31) were also substantially reduced in Group 3, suggesting reduced neovascularization following VEGF receptor inhibition (Figure 6I,J). These results conclusively demonstrate that blocking pathological angiogenesis effectively mitigates IVDD progression.

## 4 Discussion

A causal relationship exists between aging and IVDD, wherein progressive age‐related degenerative changes occur in disc structure and function (Colombier et al. 2014). During degeneration, NP exhibits cellular senescence and dysregulates ECM metabolism, AF exhibits collagen fiber degeneration with fissure formation, and cartilaginous endplates undergo calcification with reduced permeability. However, the key molecular mechanisms regulating these processes remain incompletely understood (L. Li, Zhang, et al. 2025). These pathological changes ultimately manifest as catabolism‐dominant metabolic imbalance, ECM degradation, decreased tissue hydration, fibrosis, the presence of pro‐inflammatory mediators, and diminished intervertebral height (Francisco et al. 2022; Molinos et al. 2015). The metabolism and phenotypes of NP and AF cells are critical determinants of IVDD health and disease, and metabolic imbalances inevitably induce biochemical alterations. Through broad‐spectrum biochemical profiling of IVD tissues from aged and young patients, we identified significant alterations in metabolites, including MMA. Previous studies have indicated that MMA metabolism is highly dependent on healthy mitochondria and mitochondrial enzymes (Green et al. 2017; Wang et al. 2022). MMA exacerbates aging by impairing the mitochondrial respiratory chain, thereby inducing mitochondrial dysfunction and oxidative stress (Wang et al. 2020; Stepien et al. 2017). To date, no studies have reported the relationship between MMA and IVDD. Our metabolite analysis revealed the presence of MMA within the IVD, with significantly higher concentrations in aged tissues than in young tissues. Given that MMA induces cellular senescence, which is closely associated with IVDD, we hypothesized that MMA contributes to the pathogenesis of IVDD. Subsequent in vivo and in vitro experiments confirmed this hypothesis.

To investigate the specific mechanism by which MMA induces IVDD, we performed RNA sequencing, identifying *CCL7* as a key molecule. CCL7, a member of the CC chemokine family, is a small cytokine secreted by various cell types that recruit immune cells, such as monocytes and macrophages, to inflammatory sites, thereby exacerbating local inflammation. Degenerated IVDs form an inflammatory microenvironment containing multiple cytokines, including CCL2 and CCL7 (Phillips et al. 2013; Risbud and Shapiro 2014). Our experimental results demonstrated significantly elevated CCL7 expression in aged human IVDs, consistent with the findings of Phillips et al. (2015), who reported that CCL7 expression markedly increases in degenerated discs and correlates with degeneration severity (Phillips et al. 2015; Wang et al. 2013). MMA intervention in NPCs substantially upregulates CCL7 expression. Following *CCL7* knockdown, disc tissues exhibited significantly reduced MMP3 and MMP13 levels, increased COL2 and ACAN expression, restored ECM homeostasis, and markedly delayed IVDD progression, further confirming the pivotal role of CCL7 in disc degeneration. Mechanistic exploration revealed that CCL7 primarily binds to its receptors CCR1, CCR2, and CCR3. Although these receptors do not directly activate the JAK–STAT pathway, the core function of CCL7 involves recruiting receptor‐expressing immune cells, particularly monocytes and macrophages, to promote the release of inflammatory factors. This creates a local, high‐concentration milieu of JAK2‐STAT3 activators, thereby indirectly activating the JAK2‐STAT3 pathway (Chen et al. 2025; Xie et al. 2021). Activated JAK induces STAT phosphorylation and dimerization, followed by nuclear translocation and the regulation of gene transcription (Samra et al. 2025). Pathway activation promotes the production of catabolic factors such as cyclooxygenase‐2 (i.e., COX‐2) and MMP13 in AF cells, thereby exacerbating IVDD (Suzuki et al. 2017). In this study, treatment with the STAT3 phosphorylation inhibitor, Stattic, significantly reduced MMP3, MMP13, and VEGF expression and upregulated ACAN and COL2 expression by inhibiting STAT3 phosphorylation, ultimately attenuating IVDD progression.

Traditionally, IVDs are considered the largest avascular organ in the human body (Waxenbaum et al. 2025). In normal adult IVDs, only the outer annulus fibrosus (AF) contains sparse microvessels, and the nucleus pulposus (NP) and inner AF rely entirely on diffusion from the microvasculature of the cartilaginous endplates or outer AF to obtain nutrients (e.g., oxygen and glucose). This avascular state is fundamental for maintaining low immunogenicity and matrix homeostasis in the IVDs (Waxenbaum et al. 2025). IVD has adapted to this unique microenvironment without a direct oxygen supply. When abnormal vascularization occurring within the inner AF or NP, referred to as “disc vascularization”. Although the abnormal growth of blood vessels can provide some nutrients, it simultaneously causes immune cells and inflammatory factors to infiltrate NPCs, thereby destroying their immune privilege (S. P. Zhang et al. 2025). These cells produce abundant growth factors and cytokines that alter the internal disc microenvironment, which impairs NP cell function, disturbs ECM homeostasis and accelerates IVDD (Apte et al. 2019; Zhang et al. 2022). Disc vascularization also alters the oxygen concentration in NPCs, changing the hypoxic microenvironment of IVD and influencing the phenotype and function of NPCs (Sun et al. 2021). This vascular ingrowth establishes connections between the NP and the systemic circulation, disrupting the local microenvironment and creating an oxygen‐rich state that impairs the synthesis of collagen II and aggrecan. Moreover, vascular ingrowth is accompanied by nerve proliferation, with pain‐related neurotransmitters, such as substance P and CGRP, detectable around the neo‐vessels, potentially contributing to degenerative low back pain (Freemont et al. 2002; La Binch et al. 2014). Therefore, this phenomenon does not represent physiological repair but rather a pathological response reflecting an imbalance in the tissue microenvironment during degeneration, commonly observed in the middle to late stages of IVDD (Han et al. 2025; Sun et al. 2021; Swahn et al. 2024; Tang et al. 2024). Current evidence identifies neovascularization as a pivotal pathological feature of disc degeneration, providing entry routes for immune cells, inflammatory factors, and MMPs in NP tissues, thereby accelerating degeneration. Thus, intradiscal neovascularization is a key hallmark of IVDD (Ge et al. 2022). In conclusion, disc vascularization is regarded as the crucial initial pathological step of IVDD and is a key pathological alteration in IVDD.

VEGF is a key regulatory factor for angiogenesis, directly influencing the activity, proliferation, and migration of vascular endothelial cells (Lee et al. 2025). VEGF expression in IVDD is closely correlated with disease severity, and elevated VEGF levels signify the onset of degeneration (Si Ping Zhang et al. 2025). Our research has revealed that the expression of VEGF within NPCs of elderly individuals is higher, and the number of CD31‐labeled blood vessels is also greater. Elevated VEGF expression was observed in both elderly human discs and MMA‐treated NPCs. RNA sequencing of NP tissues has further implicated vascularization in the pathogenesis of IVDD. Therefore, we believe that MMA promotes disc vascularization by increasing VEGF expression, ultimately accelerating IVDD. Our research indicates that VEGF expression is associated with the CCL7/JAK2‐STAT3 signaling pathway. This is consistent with the reports by many previous scholars that JAK2‐STAT3 promotes angiogenesis and increases vascular permeability through VEGF (Mantsounga et al. 2022; Wang et al. 2021; Chen et al. 2023). Ouyang et al. also demonstrated that activation of this signaling axis also promotes the proliferation and migration of vascular endothelial cells (Ouyang et al. 2021). Therefore, we believe that during IVDD progression, the activated JAK2/STAT3 signaling enhances endothelial cell activation, driving pathological neovascularization within the NPCs, and ultimately leads to disc vascularization. The reduction in VEGF and CD31 expression both in vitro and in vivo after using Stattic supports our viewpoint. In order to demonstrate the adverse effects of disc vascularization on the IVD, we used the broad‐spectrum VEGF receptor inhibitor, lenvatinib, to block vascularization in vivo. The results demonstrated that treatment with MMA + lenvatinib substantially reduced vascularization, retained a greater amount of the NPCs, and improved the disc degeneration metrics compared to the MMA‐only treatment. This ultimately proves that disc vascularization is harmful to IVDD and is an important pathological change of IVDD. Inhibiting disc vascularization can alleviate the severity of intervertebral disc degeneration, and anti‐vertebral disc vascularization therapy is a key target for treating IVDD.

We propose that MMA accumulation in senescent NPCs and aging IVD disrupts the balance between ECM synthesis and catabolism, shifting the equilibrium toward catabolic dominance. Pathological microvascular invasion driven by JAK2/STAT3 pathway activation accelerates metabolic dysregulation and structural disintegration, culminating in progressive IVD functional impairment that accelerates IVDD. This cascade amplifies VEGF expression within degenerating NP cells, triggering vascular endothelial cell activation and accelerating pathological microvascular formation. Subsequent vascularization of the disc led to an increase in the infiltration of immune cells and the accumulation of inflammatory factors, which exacerbated the inflammation, altered the microenvironment within the intervertebral disc, and accelerated the degeneration of the intervertebral disc.

This study has some limitations: when verifying the effect of MMA on IVDD in mice, the injection administration method has certain limitations. However, based on X‐ray, MRI, and tissue staining results, it still proves that MMA can accelerate intervertebral disc degeneration in vivo and still has certain reliability. IVDD is a complex process, involving multiple energy metabolism or aging‐related pathways. This study focused on aging‐related pathways and IVDD. In future research, we will explore other related changes in energy metabolism under IVDD conditions. Although we achieved the goal of alleviating intervertebral disc degeneration with lenvatinib, it is relatively difficult to achieve clinical transformation. In the future, we will combine nanomedicine delivery systems or hydrogels as carrier materials to achieve local precise, controllable sustained release, and long‐term effective goals, accelerating clinical transformation. While male mice were used to minimize hormonal variability, we recognize this as a significant limitation. Sex is a pivotal variable influencing disc immunity and degeneration (Clayton et al. 2025); thus, the MMA‐induced pathways identified here may exhibit sexual dimorphism requiring future validation in female models. In addition to these biological variables, the mouse caudal model lacks the physiological loading of the human lumbar spine and is subject to age‐related vertebral elongation, factors that may alter degeneration rates and phenotypes. Consequently, future studies using lumbar or dynamic loading models are necessary to confirm these metabolic interactions under site‐specific stressors. Importantly, despite standardized sectioning, severe disc degeneration inherently alters anatomical landmarks. The flattened endplate curvature in MMA groups, likely secondary to disc height and NP volume loss, represents a morphological consequence of degeneration that should be accounted for in histological assessments.

Our study conclusively demonstrates that MMA drives IVDD progression by promoting disc vascularization and delineates its underlying molecular cascade. By targeting this pathway, we propose a focused therapeutic strategy, thereby opening a novel avenue for treatment. Despite certain limitations, the key findings of this work innovatively address a significant knowledge gap in the field, providing mechanistic insights and a potential intervention target for IVDD.

## Author Contributions

Bin Zhao and Doudou Jing designed the research. Yuanzhang Jin, Runtian Zhou, and Xiaonan Wang performed the experiments. Yuanzhang Jin, Haifeng Liu, and Xiaofeng Zhao collected data and performed image analysis experiments. Yuanzhang Jin wrote the original manuscript.

## Funding

This work was supported by the Youth Project of Shanxi Basic Research Program [202203021222389], the Shanxi Province Science and Technology Achievement Transformation Guidance Special Project [202304021301064] and the Shanxi Province Basic Research Program [202203021221274].

## Ethics Statement

This study was approved by the Ethics Committee of the Second Hospital of Shanxi Medical University. (No. [2025] YX 050). All procedures were conducted following the Declaration of Helsinki, and all participants provided informed consent.

## Conflicts of Interest

The authors declare no conflicts of interest.

## Supporting information


**Data S1:** acel70436‐sup‐0001‐Figures.docx.

**Table S1:** Antibody usage.

## Data Availability

All data that support the findings of this study are available to the researchers on reasonable request.

## References

[acel70436-bib-0001] Apte, R. S. , D. S. Chen , and N. Ferrara . 2019. “VEGF in Signaling and Disease: Beyond Discovery and Development.” Cell 176, no. 6: 1248–1264. 30849371 10.1016/j.cell.2019.01.021 PMC6410740

[acel70436-bib-0002] Bhujel, B. , H. E. Shin , D. J. Choi , and I. Han . 2022. “Mesenchymal Stem Cell‐Derived Exosomes and Intervertebral Disc Regeneration: Review.” International Journal of Molecular Sciences 23, no. 13: 7306. 10.3390/ijms23137306. 35806304 PMC9267028

[acel70436-bib-0003] Cao, B. , Y. Xue , and D. Liu . 2024. “The Association Between Methylmalonic Acid, a Biomarker of Mitochondria Dysfunction, and Phenotypic Age Acceleration: A Population‐Based Study.” Archives of Gerontology and Geriatrics 117: 105176. 10.1016/j.archger.2023.105176. 37713936

[acel70436-bib-0004] Chen, J. , S. Shi , X. Li , et al. 2025. “CCL7 Promotes Macrophage Polarization and Synovitis to Exacerbate Rheumatoid Arthritis.” iScience 28, no. 4: 112177. 10.1016/j.isci.2025.112177. 40224025 PMC11987677

[acel70436-bib-0005] Chen, S. , J. Zhang , D. Sun , et al. 2023. “SYVN1 Promotes STAT3 Protein Ubiquitination and Exerts Antiangiogenesis Effects in Retinopathy of Prematurity Development.” Investigative Ophthalmology & Visual Science 64, no. 11: 12. 10.1167/iovs.64.11.8 PMC10408771 37540175

[acel70436-bib-0006] Chen, X. , A. Zhang , K. Zhao , et al. 2024. “The Role of Oxidative Stress in Intervertebral Disc Degeneration: Mechanisms and Therapeutic Implications.” Ageing Research Reviews 98: 102323. 10.1016/j.arr.2024.102323. 38734147

[acel70436-bib-0007] Clayton, S. W. , R. E. Walk , L. Mpofu , G. W. D. Easson , and S. Y. Tang . 2025. “Sex‐Specific Divergences in the Types and Timing of Infiltrating Immune Cells During the Intervertebral Disc Acute Injury Response and Their Associations With Degeneration.” Osteoarthritis and Cartilage 33, no. 2: 247–260. 10.1016/j.joca.2024.10.002. 39426787 PMC12525793

[acel70436-bib-0008] Colombier, P. , J. Clouet , O. Hamel , L. Lescaudron , and J. M. Guicheux . 2014. “The Lumbar Intervertebral Disc: From Embryonic Development to Degeneration.” Joint Bone Spine 81, no. 2: 125–129. 23932724 10.1016/j.jbspin.2013.07.012

[acel70436-bib-0009] Costa, R. T. , M. B. Santos , C. Alberto‐Silva , D. C. Carrettiero , and C. A. J. Ribeiro . 2023. “Methylmalonic Acid Impairs Cell Respiration and Glutamate Uptake in C6 Rat Glioma Cells: Implications for Methylmalonic Acidemia.” Cellular and Molecular Neurobiology 43, no. 3: 1163–1180. 10.1007/s10571-022-01236-1. 35674974 PMC11414442

[acel70436-bib-0010] Curic, G. 2020. “Intervertebral Disc and Adipokine Leptin‐Loves Me, Loves Me Not.” International Journal of Molecular Sciences 22, no. 1: 375. 10.3390/ijms22010375. 33396484 PMC7795371

[acel70436-bib-0011] de Souza Almeida, R. R. , L. D. Bobermin , B. Parmeggiani , et al. 2022. “Methylmalonic Acid Induces Inflammatory Response and Redox Homeostasis Disruption in C6 Astroglial Cells: Potential Glioprotective Roles of Melatonin and Resveratrol.” Amino Acids 54, no. 11: 1505–1517. 10.1007/s00726-022-03191-z. 35927507

[acel70436-bib-0012] Farag, M. , R. Rezk , H. Hutchinson , A. Zankevich , and B. Lucke‐Wold . 2024. “Intervertebral Disc Degeneration and Regenerative Medicine.” Clinical and Translational Discovery 4, no. 3: e289. 10.1002/ctd2.289.

[acel70436-bib-0013] Feng, Y. , B. Egan , and J. Wang . 2016. “Genetic Factors in Intervertebral Disc Degeneration.” Genes and Diseases 3, no. 3: 178–185. 10.1016/j.gendis.2016.04.005. 27617275 PMC5016799

[acel70436-bib-0014] Francisco, V. , J. Pino , M. á. González‐Gay , et al. 2022. “A New Immunometabolic Perspective of Intervertebral Disc Degeneration.” Nature Reviews Rheumatology 18, no. 1: 47–60. 34845360 10.1038/s41584-021-00713-z

[acel70436-bib-0015] Freemont, A. J. , A. Watkins , C. Le Maitre , et al. 2002. “Nerve Growth Factor Expression and Innervation of the Painful Intervertebral Disc.” Journal of Pathology 197, no. 3: 286–292. 10.1002/path.1108. 12115873

[acel70436-bib-0016] Ge, Y. , Y. Chen , C. Guo , et al. 2022. “Pyroptosis and Intervertebral Disc Degeneration: Mechanistic Insights and Therapeutic Implications.” Journal of Inflammation Research 15: 5857–5871. 10.2147/jir.S382069. 36263145 PMC9575467

[acel70436-bib-0017] Gomes, A. P. , D. Ilter , V. Low , et al. 2020. “Age‐Induced Accumulation of Methylmalonic Acid Promotes Tumour Progression.” Nature 585, no. 7824: 283–287. 10.1038/s41586-020-2630-0. 32814897 PMC7785256

[acel70436-bib-0018] Green, R. , L. H. Allen , A. L. Bjørke‐Monsen , et al. 2017. “Vitamin B(12) Deficiency.” Nature Reviews. Disease Primers 3: 17040. 10.1038/nrdp.2017.40. 28660890

[acel70436-bib-0019] Griffith, J. F. , Y. X. Wang , G. E. Antonio , et al. 2007. “Modified Pfirrmann Grading System for Lumbar Intervertebral Disc Degeneration.” Spine (Phila Pa 1976) 32, no. 24: E708–E712. 10.1097/BRS.0b013e31815a59a0. 18007231

[acel70436-bib-0020] Han, L. , F. Li , H. Wu , et al. 2025. “Targeting FABP4 to Inhibit AGEs‐RAGE/NF‐κB Signalling Effectively Ameliorates Nucleus Pulposus Dysfunction and Angiogenesis in Obesity‐Related Intervertebral Disc Degeneration.” Cell Proliferation 58, no. 9: e70021. 10.1111/cpr.70021. 40090836 PMC12414640

[acel70436-bib-0021] Huang, J. , S. L. Lian , J. H. Han , Z. C. Lu , and Y. Ding . 2023. “Pure Platelet‐Rich Plasma Promotes Semaphorin‐3A Expression: A Novel Insight to Ameliorate Intervertebral Disk Degeneration In Vitro.” Journal of Orthopaedic Surgery and Research 18, no. 1: 789. 37864189 10.1186/s13018-023-04290-7 PMC10588088

[acel70436-bib-0022] Kashtan, C. E. , M. Abousedira , S. Rozen , J. C. Manivel , M. McCann , and M. Tuchman . 1998. “Chronic Administration of Methylmalonic Acid (MMA) to Rats Causes Proteinuria and Renal Tubular Injury • 1815.” Pediatric Research 43, no. 4: 309. 10.1203/00006450-199804001-01838.

[acel70436-bib-0023] La Binch, A. , A. A. Cole , L. M. Breakwell , et al. 2014. “Expression and Regulation of Neurotrophic and Angiogenic Factors During Human Intervertebral Disc Degeneration.” Arthritis Research & Therapy 16, no. 4: 416. 10.1186/s13075-014-0416-1. 25209447 PMC4177417

[acel70436-bib-0024] Lee, C. , M.‐J. Kim , A. Kumar , H.‐W. Lee , Y. Yang , and Y. Kim . 2025. “Vascular Endothelial Growth Factor Signaling in Health and Disease: From Molecular Mechanisms to Therapeutic Perspectives.” Signal Transduction and Targeted Therapy 10, no. 1: 170. 10.1038/s41392-025-02249-0. 40383803 PMC12086256

[acel70436-bib-0025] Li, F. , Y. Shi , J. Chen , et al. 2025. “LGR6 Modulates Intervertebral Disc Degeneration Through Regulation of Macrophage Efferocytosis.” Journal of Translational Medicine 23, no. 1: 475. 10.1186/s12967-025-06427-0. 40281518 PMC12023656

[acel70436-bib-0026] Li, L. , G. Zhang , Z. Yang , Z. Cao , D. Wang , and X. Kang . 2025. “STMN1–IGFBP5 Axis Induces Senescence and Extracellular Matrix Degradation in Nucleus Pulposus Cells: In Vivo and In Vitro Insights.” Molecular Medicine 31, no. 1: 167. 40319242 10.1186/s10020-025-01220-7 PMC12049776

[acel70436-bib-0027] Li, P. , R. Liao , J. Li , et al. 2025. “Stattic Engineering M2 Macrophage‐Derived Exosomes Mediate Autophagy and Immune Reprogramming for Secondary Hyperparathyroidism.” Journal of Advanced Research 81: 781–796. 10.1016/j.jare.2025.06.016. 40505957 PMC12957820

[acel70436-bib-0028] Li, Z. , V. Low , V. Luga , et al. 2022. “Tumor‐Produced and Aging‐Associated Oncometabolite Methylmalonic Acid Promotes Cancer‐Associated Fibroblast Activation to Drive Metastatic Progression.” Nature Communications 13, no. 1: 6239. 10.1038/s41467-022-33862-0. PMC9584945 36266345

[acel70436-bib-0029] Mantsounga, C. S. , C. Lee , J. Neverson , et al. 2022. “Macrophage IL‐1β Promotes Arteriogenesis by Autocrine STAT3‐ and NF‐κB‐Mediated Transcription of Pro‐Angiogenic VEGF‐A.” Cell Reports 38, no. 5: 110309. 10.1016/j.celrep.2022.110309. 35108537 PMC8865931

[acel70436-bib-0030] Matsui, J. , Y. Yamamoto , Y. Funahashi , et al. 2008. “E7080, a Novel Inhibitor That Targets Multiple Kinases, Has Potent Antitumor Activities Against Stem Cell Factor Producing Human Small Cell Lung Cancer H146, Based on Angiogenesis Inhibition.” International Journal of Cancer 122, no. 3: 664–671. 10.1002/ijc.23131. 17943726

[acel70436-bib-0031] McMurray, J. S. 2006. “A New Small‐Molecule Stat3 Inhibitor.” Chemistry & Biology 13, no. 11: 1123–1124. 10.1016/j.chembiol.2006.11.001. 17113993

[acel70436-bib-0032] Miller, J. A. , C. Schmatz , and A. B. Schultz . 1988. “Lumbar Disc Degeneration: Correlation With Age, Sex, and Spine Level in 600 Autopsy Specimens.” Spine (Phila Pa 1976) 13, no. 2: 173–178. 3406837

[acel70436-bib-0033] Molinos, M. , C. R. Almeida , J. Caldeira , C. Cunha , R. M. Goncalves , and M. A. Barbosa . 2015. “Inflammation in Intervertebral Disc Degeneration and Regeneration.” Journal of the Royal Society Interface 12, no. 104: 20141191. 25673296 10.1098/rsif.2014.1191 PMC4345483

[acel70436-bib-0034] Oscar, A. G. , M. Tokio , O. Merissa , et al. 2018. “FOXO Are Required for Intervertebral Disk Homeostasis During Aging and Their Deficiency Promotes Disk Degeneration.” Aging Cell 17: e12800. 29963746 10.1111/acel.12800 PMC6156454

[acel70436-bib-0035] Ouyang, S. , Y. Li , X. Wu , et al. 2021. “GPR4 Signaling Is Essential for the Promotion of Acid‐Mediated Angiogenic Capacity of Endothelial Progenitor Cells by Activating STAT3/VEGFA Pathway in Patients With Coronary Artery Disease.” Stem Cell Research & Therapy 12, no. 1: 149. 10.1186/s13287-021-02221-z. 33632325 PMC7905863

[acel70436-bib-0036] Phillips, K. L. , K. Cullen , N. Chiverton , et al. 2015. “Potential Roles of Cytokines and Chemokines in Human Intervertebral Disc Degeneration: Interleukin‐1 Is a Master Regulator of Catabolic Processes.” Osteoarthritis and Cartilage 23, no. 7: 1165–1177. 25748081 10.1016/j.joca.2015.02.017

[acel70436-bib-0037] Phillips, K. L. E. , N. Chiverton , A. L. Michael , et al. 2013. “The Cytokine and Chemokine Expression Profile of Nucleus Pulposus Cells: Implications for Degeneration and Regeneration of the Intervertebral Disc.” Arthritis Research & Therapy 15, no. 6: R213. 24325988 10.1186/ar4408 PMC3979161

[acel70436-bib-0038] Rannou, F. , T. S. Lee , R. H. Zhou , et al. 2004. “Intervertebral Disc Degeneration: The Role of the Mitochondrial Pathway in Annulus Fibrosus Cell Apoptosis Induced by Overload.” American Journal of Pathology 164, no. 3: 915–924. 10.1016/s0002-9440(10)63179-3. 14982845 PMC1613264

[acel70436-bib-0039] Risbud, M. V. , and I. M. Shapiro . 2014. “Role of Cytokines in Intervertebral Disc Degeneration: Pain and Disc Content.” Nature Reviews Rheumatology 10, no. 1: 44–56. 24166242 10.1038/nrrheum.2013.160 PMC4151534

[acel70436-bib-0040] Samra, S. , J. R. E. Bergerson , A. F. Freeman , and S. E. T. Dphil . 2025. “JAK‐STAT Signaling Pathway, Immunodeficiency, Inflammation, Immune Dysregulation, and Inborn Errors of Immunity.” Journal of Allergy and Clinical Immunology 155, no. 2: 357–367. 39369964 10.1016/j.jaci.2024.09.020

[acel70436-bib-0041] Shafer, M. , V. Low , Z. Li , and J. Blenis . 2025. “The Emerging Role of Dysregulated Propionate Metabolism and Methylmalonic Acid in Metabolic Disease, Aging, and Cancer.” Cell Metabolism 37, no. 2: 316–329. 10.1016/j.cmet.2025.01.005. 39908986 PMC11984558

[acel70436-bib-0042] Stepien, K. M. , R. Heaton , S. Rankin , et al. 2017. “Evidence of Oxidative Stress and Secondary Mitochondrial Dysfunction in Metabolic and Non‐Metabolic Disorders.” Journal of Clinical Medicine 6, no. 7: 71. 10.3390/jcm6070071. 28753922 PMC5532579

[acel70436-bib-0043] Sun, Z. , H. Zhao , B. Liu , et al. 2021. “AF Cell Derived Exosomes Regulate Endothelial Cell Migration and Inflammation: Implications for Vascularization in Intervertebral Disc Degeneration.” Life Sciences 265: 118778. 10.1016/j.lfs.2020.118778. 33217442

[acel70436-bib-0044] Suzuki, S. , N. Fujii , T. Watanabe , et al. 2017. “Potential Involvement of the IL‐6/JAK/STAT3 Pathway in the Pathogenesis of Intervertebral Disc Degeneration.” Spine 42, no. 14: E817–E824. 27879577 10.1097/BRS.0000000000001982

[acel70436-bib-0045] Swahn, H. , J. Mertens , M. Olmer , et al. 2024. “Shared and Compartment‐Specific Processes in Nucleus Pulposus and Annulus Fibrosus During Intervertebral Disc Degeneration.” Advanced Science (Weinh) 11, no. 17: e2309032. 10.1002/advs.202309032. PMC11077672 38403470

[acel70436-bib-0046] Tang, F. , M. Yan , Z. Wang , et al. 2025. “Mitochondrial Metabolite Methylmalonic Acid, Subclinical Myocardial Injury, and Its Incremental Predictive Value for Cardiovascular Mortality Risk.” Archives of Medical Research 56, no. 6: 103226. 40347706 10.1016/j.arcmed.2025.103226

[acel70436-bib-0047] Tang, J. , Y. Luo , Q. Wang , J. Wu , and Y. Wei . 2024. “Stimuli‐Responsive Delivery Systems for Intervertebral Disc Degeneration.” International Journal of Nanomedicine 19: 4735–4757. 10.2147/ijn.S463939. 38813390 PMC11135562

[acel70436-bib-0048] Wang, J. , Y. Tian , K. L. Phillips , et al. 2013. “Tumor Necrosis Factor α‐ and Interleukin‐1β‐Dependent Induction of CCL3 Expression by Nucleus Pulposus Cells Promotes Macrophage Migration Through CCR1.” Arthritis & Rheumatism 65, no. 3: 832–842. 10.1002/art.37819. 23233369 PMC3582738

[acel70436-bib-0049] Wang, L. , M. Astone , S. K. Alam , et al. 2021. “Suppressing STAT3 Activity Protects the Endothelial Barrier From VEGF‐Mediated Vascular Permeability.” Company of Biologists 11: dmm049029. 10.1242/dmm.049029 PMC8592016 34542605

[acel70436-bib-0050] Wang, S. , Y. Liu , J. Liu , et al. 2020. “Mitochondria‐Derived Methylmalonic Acid, a Surrogate Biomarker of Mitochondrial Dysfunction and Oxidative Stress, Predicts All‐Cause and Cardiovascular Mortality in the General Population.” Redox Biology 37: 101741. 10.1016/j.redox.2020.101741. 33035815 PMC7554255

[acel70436-bib-0051] Wang, S. , Y. Wang , X. Wan , et al. 2022. “Cobalamin Intake and Related Biomarkers: Examining Associations With Mortality Risk Among Adults With Type 2 Diabetes in NHANES.” Diabetes Care 45, no. 2: 276–284. 10.2337/dc21-1674. 34862259 PMC8914415

[acel70436-bib-0052] Waxenbaum, J. A. , V. Reddy , and B. Futterman . 2025. “Anatomy, Back, Intervertebral Discs.” In StatPearls. StatPearls Publishing Copyright, StatPearls Publishing LLC.

[acel70436-bib-0053] Wen, Z. Q. , J. Lin , and Y. S. Xie . 2023. “Insights Into the Underlying Pathogenesis and Therapeutic Potential of Endoplasmic Reticulum Stress in Degenerative Musculoskeletal Diseases.” Military Medical Research 10, no. 1: 54. 37941072 10.1186/s40779-023-00485-5 PMC10634069

[acel70436-bib-0054] Wu, O. , Y. Jin , Z. Zhang , et al. 2024. “KMT2A Regulates the Autophagy‐GATA4 Axis Through METTL3‐Mediated m(6)A Modification of ATG4a to Promote NPCs Senescence and IVDD Progression.” Bone Research 12, no. 1: 67. 10.1038/s41413-024-00373-1. 39572532 PMC11582572

[acel70436-bib-0055] Xia, Q. , Y. Zhao , H. Dong , et al. 2024. “Progress in the Study of Molecular Mechanisms of Intervertebral Disc Degeneration.” Biomedicine & Pharmacotherapy 174: 116593. 10.1016/j.biopha.2024.116593. 38626521

[acel70436-bib-0056] Xie, C. , F. Ye , N. Zhang , Y. Huang , Y. Pan , and X. Xie . 2021. “CCL7 Contributes to Angiotensin II‐Induced Abdominal Aortic Aneurysm by Promoting Macrophage Infiltration and Pro‐Inflammatory Phenotype.” Journal of Cellular and Molecular Medicine 25, no. 15: 7280–7293. 10.1111/jcmm.16757. 34189838 PMC8335673

[acel70436-bib-0057] Yokota, T. , K. Omachi , M. A. Suico , et al. 2018. “STAT3 Inhibition Attenuates the Progressive Phenotypes of Alport Syndrome Mouse Model.” Nephrology, Dialysis, Transplantation 33, no. 2: 214–223. 10.1093/ndt/gfx246. 28992339

[acel70436-bib-0058] Zhang, S. , W. Liu , S. Chen , et al. 2022. “Extracellular Matrix in Intervertebral Disc: Basic and Translational Implications.” Cell and Tissue Research 390, no. 1: 1–22. 35792910 10.1007/s00441-022-03662-5

[acel70436-bib-0059] Zhang, S. P. , M. Tong , J. Mo , Z. Y. Dong , and Y. F. Huang . 2025. “M2 Macrophages Activate the IL‐10/JAK2/STAT3 Pathway to Induce Pathological Microangiogenesis in the Nucleus Pulposus Exacerbating Intervertebral Disc Degeneration.” Journal of Orthopaedic Surgery and Research 20, no. 1: 532. 40426248 10.1186/s13018-025-05962-2 PMC12117970

[acel70436-bib-0060] Zhou, R. , X. Wang , Y. Jin , et al. 2025. “Mechanism of Lidocaine‐Induced ROS Generation Triggering DNA Double‐Strand Breaks and Promoting Intervertebral Disc Cell Senescence via the MYC‐DUSP1‐P53 Axis.” Free Radical Biology & Medicine 240: 457–471. 10.1016/j.freeradbiomed.2025.08.049. 40850532

