# Supplementary material for: Methylmalonic Acid, an Aging‐Associated Metabolite, Accelerates Intervertebral Disc Degeneration by Inducing Disc Vascularization via the CCL7/JAK2‐STAT3/VEGF Signaling Axis
Source: Aging Cell. 2026 Mar 7;25(3):e70436. doi: 10.1111/acel.70436 (PMC12967621; doi:10.1111/acel.70436)

**Fig.S1**

**A**.Representative lumbar spine MRI (T2-weighted sagittal view) of young and elderly patients submitted for metabolomic analysis (Fig 1F).

**B**.The isotype control of the IHC staining in Fig 1B.

**C**. Principal component analysis (PCA) plot derived from metabolomic detection.

**D**. Structural formulae of MMA and succinic acid.

**E**. Comparative box plot of vitamin B12 content in IVD tissues between young and elderly patients


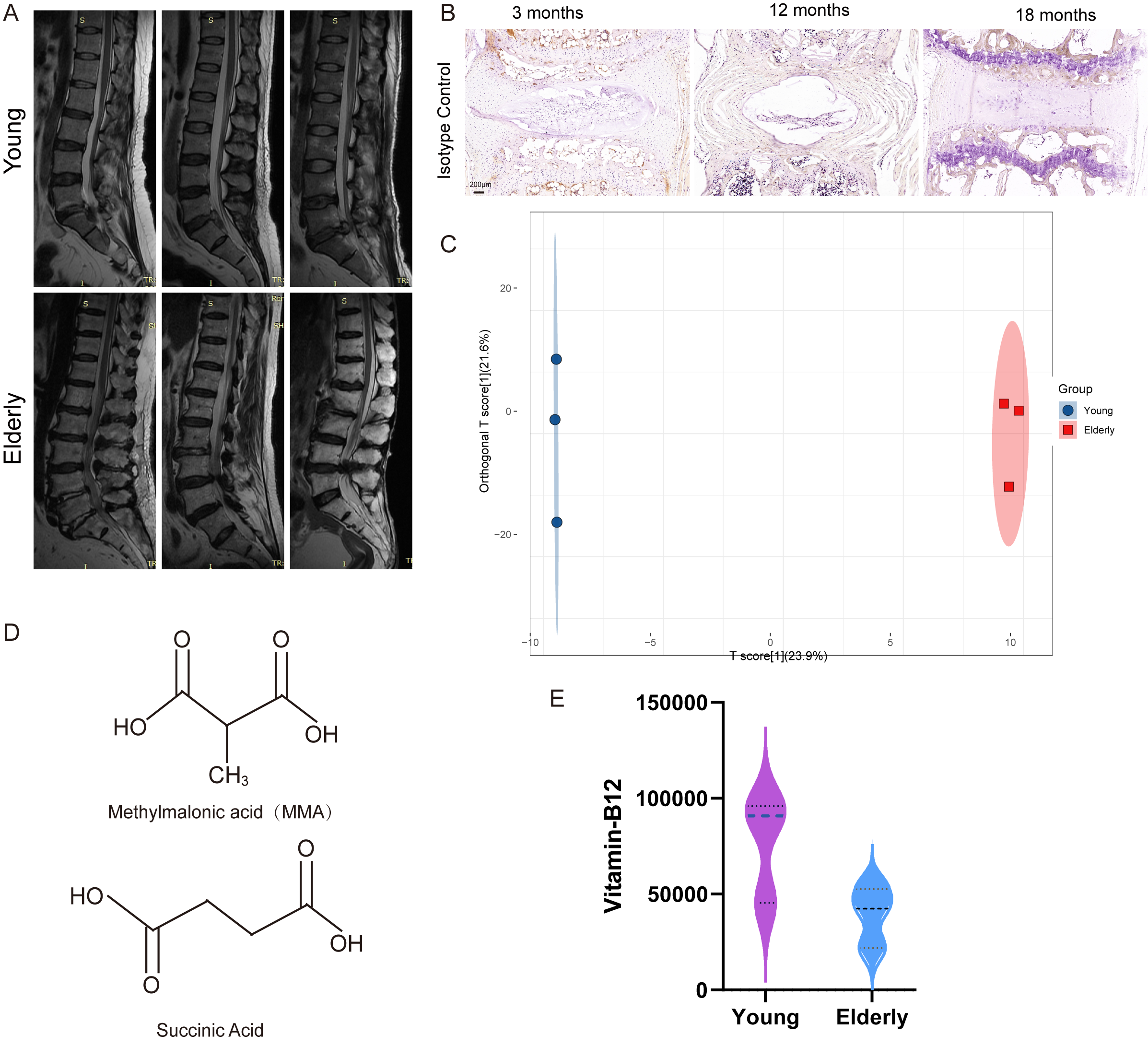


**Fig.S2**

**A**. The isotype control of the IHC staining in Fig 2I.

**B-C**. Statistical analysis of the percentage of ACAN-positive and MMP3-positive cells in Fig 2I.


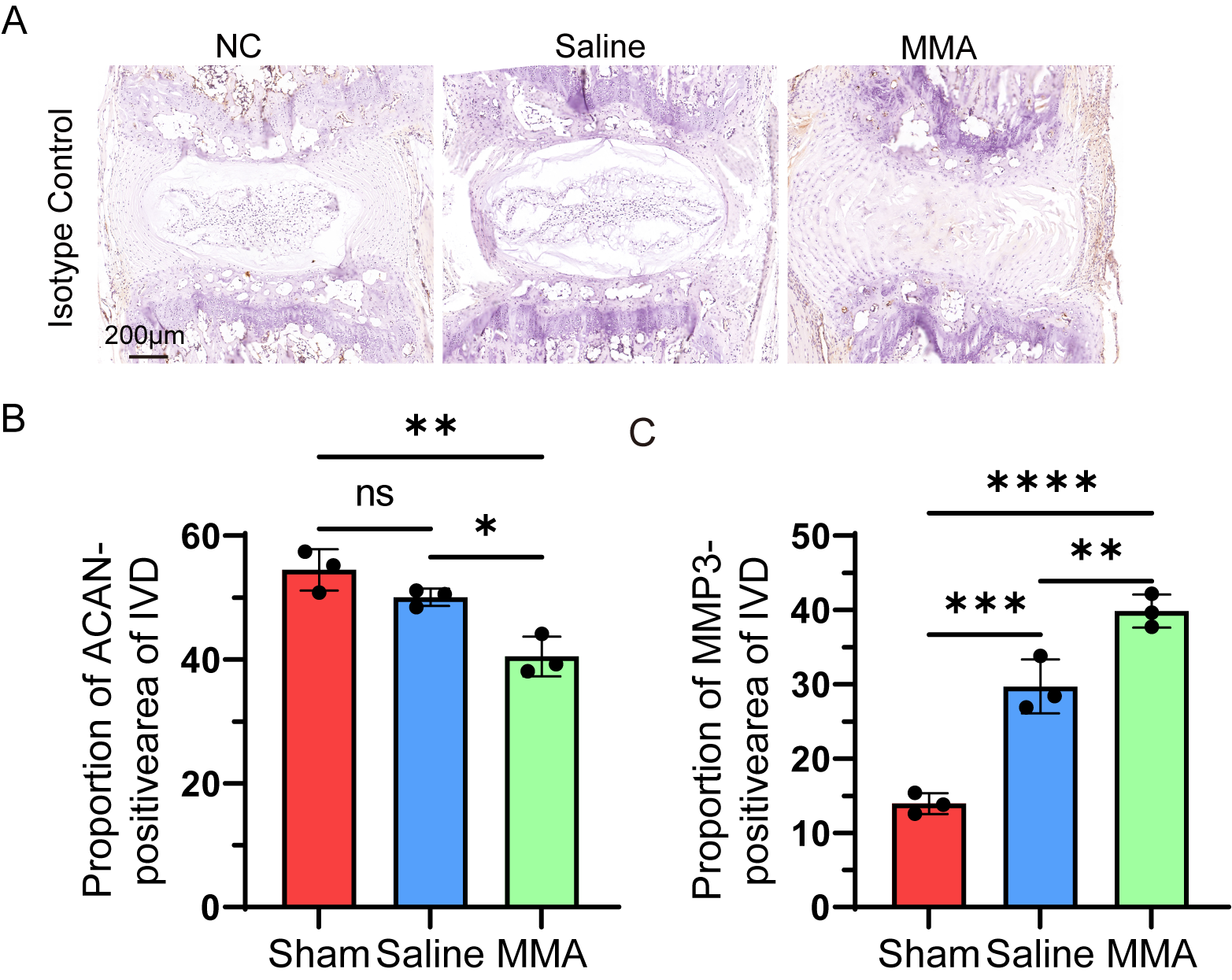


**Fig.S3**

**A**. Heatmap of DEGs.

**B**. Cell viability of NPCs treated with 0-10 μM Stattic for 24 h and 48 h was determined using the CCK-8 assay.


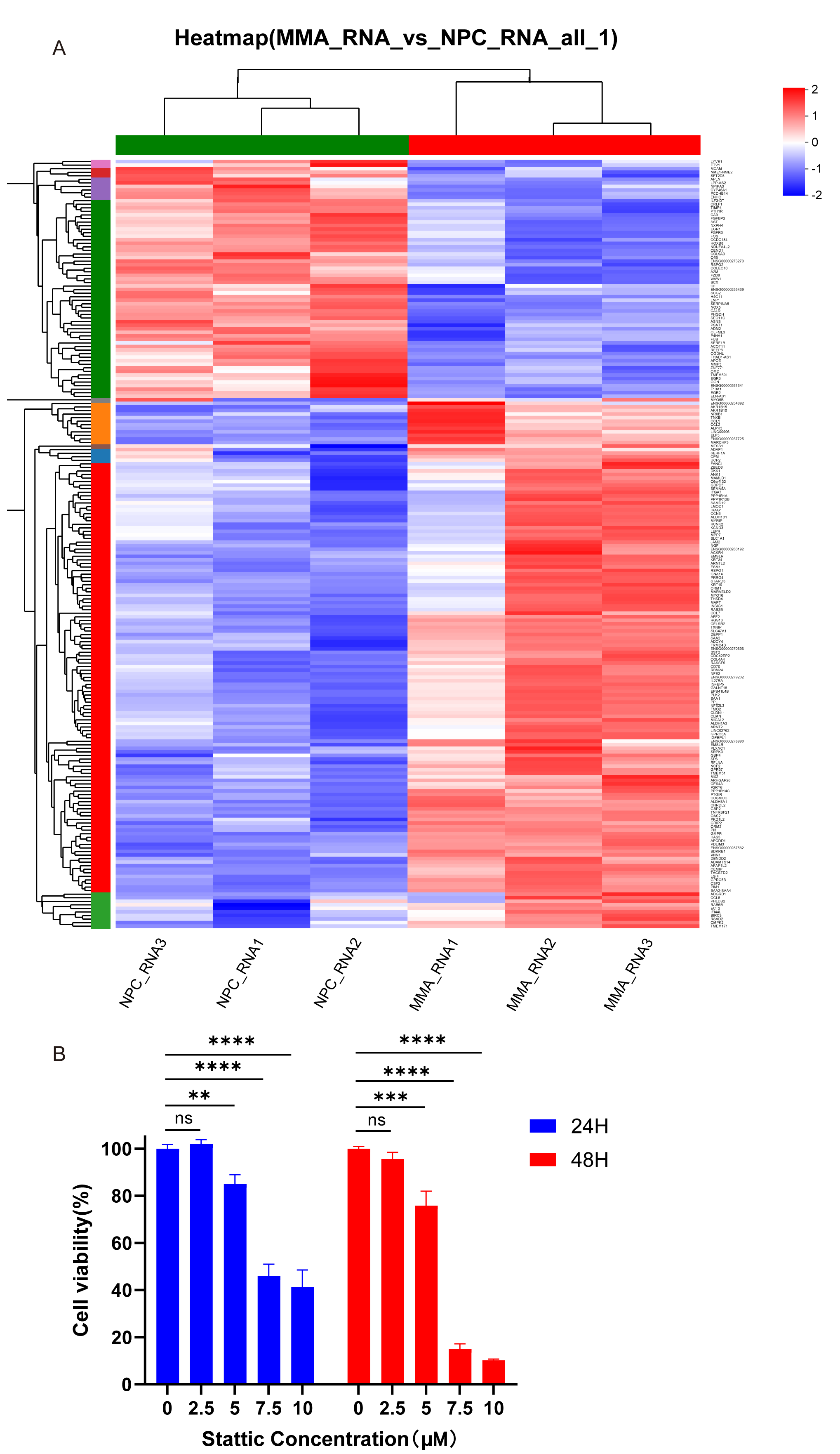


**Fig.S4**

**A**.The isotype control of the IHC staining in Fig 4N.

**B**.Quantitative analysis of Western blot results shown in Fig 5B.

**C**.Quantitative analysis of Western blot results shown in Fig 5C.

**D-E**: Statistical analysis of the percentage of VEGF-positive and CD31-positive cells in Figure 5D.

**F-H**. Representative IHC staining of VEGF and CD31 in caudal IVDs from 3-, 12-, and 18-month-old mice.Statistical analysis of the percentage of VEGF-positive and CD31-positive cells.


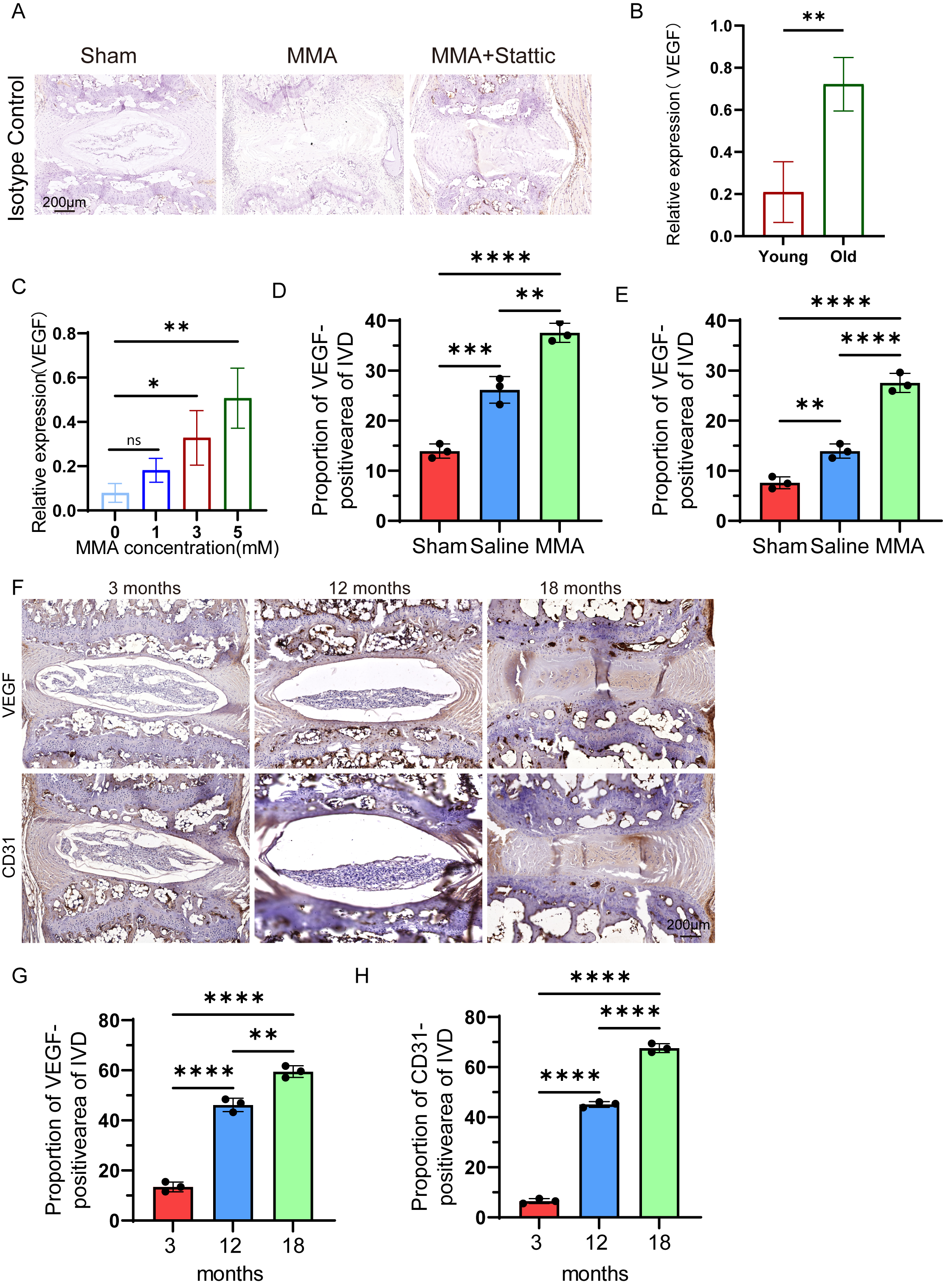


**Fig.S5**

**A**.Migration ability of HUVES treated with conditioned media from NPCs exposed to NC or 5 mM MMA assessed by scratch-wound healing assay. A scale bar of 200 μm (shown in red) applies to all images.

**B.**Quantitative statistics of the migration rate in the scratch assay.

**C.**Angiogenic potential of HUVES treated with conditioned media from NPCs exposed to NC or 5 mM MMA, evaluated using tube formation assay.White bar represents 200 μm.

**DE**.Quantitative statistics of the tube branch points and length of in the tube formation assays.


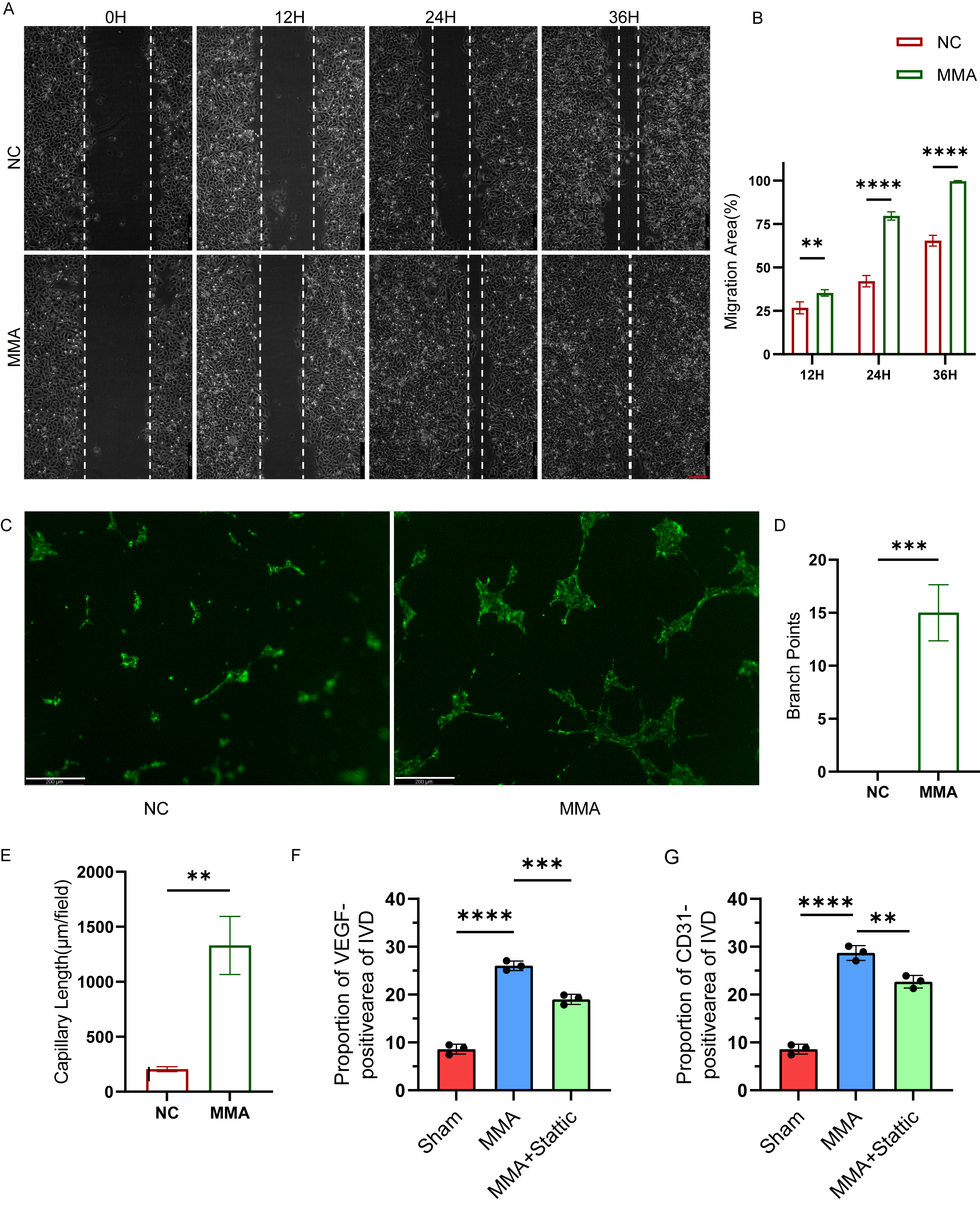


**Fig.S6**

**A.** The isotype control of the IHC staining in Fig 6E.

**B.** Biological safety analysis. Results of HE staining of liver, heart, spleen, lung and kidney sections in sham, MMA ,Static + MMA, and MMA +Lenvatinib.


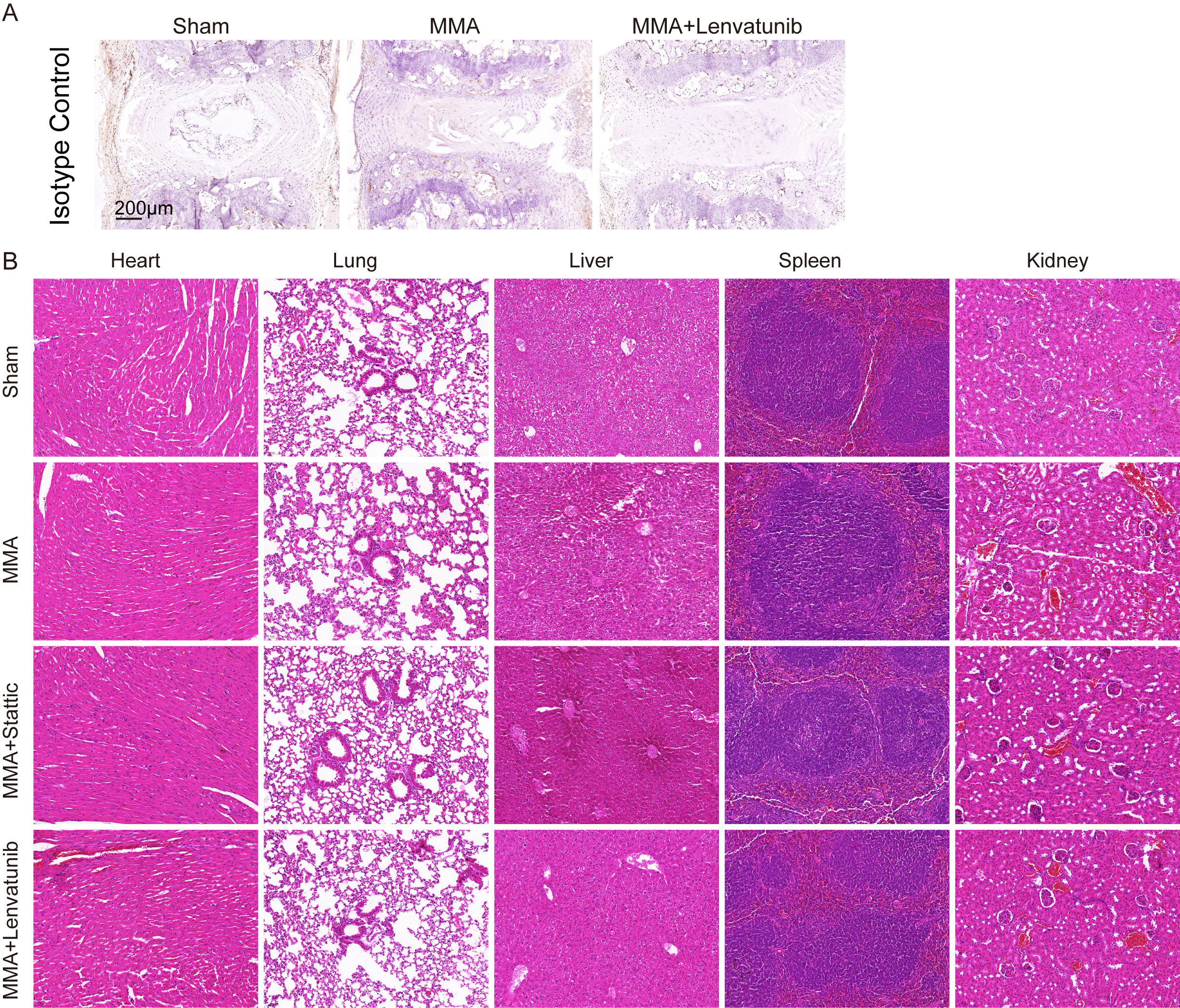

Supplement: Supplementary file 1 — Data S1: acel70436‐sup‐0001‐Figures.docx. [file ACEL-25-e70436-s002.docx]
